# The aetiology and antibiotic management of community-acquired pneumonia in adults in Europe: a literature review

**DOI:** 10.1007/s10096-014-2067-1

**Published:** 2014-02-15

**Authors:** A. Torres, F. Blasi, W. E. Peetermans, G. Viegi, T. Welte

**Affiliations:** 1 Servei de Pneumologia, Hospital Clínic de Barcelona, Institut d’Investigacions Biomèdiques August Pi i Sunyer (IDIBAPS), CIBER de Enfermedades Respiratorias (CIBERes), University of Barcelona, Barcelona, Spain; 2 Department of Pathophysiology and Transplantation, University of Milan, IRCCS Fondazione Ca’ Granda Ospedale Maggiore, Milan, Italy; 3 Department of Internal Medicine, University Hospital, KU Leuven, Leuven, Belgium; 4 CNR Institute of Clinical Physiology, Pisa, and CNR Institute of Biomedicine and Molecular Immunology, Palermo, Italy; 5 Department of Respiratory Medicine, Medizinische Hochschule, Hannover, Germany

## Abstract

The purpose of this paper was to generate up-to-date information on the aetiology of community-acquired pneumonia (CAP) and its antibiotic management in adults across Europe. Structured searches of PubMed identified information on the aetiology of CAP and its antibiotic management in individuals aged >15 years across Europe. We summarise the data from 33 studies published between January 2005 and July 2012 that reported on the pathogens identified in patients with CAP and antibiotic treatment in patients with CAP. *Streptococcus pneumoniae* was the most commonly isolated pathogen in patients with CAP and was identified in 12.0–85.0 % of patients. Other frequently identified pathogens found to cause CAP were *Haemophilus influenzae*, Gram-negative enteric bacilli, respiratory viruses and *Mycoplasma pneumoniae*. We found several age-related trends: *S. pneumoniae*, *H. influenzae* and respiratory viruses were more frequent in elderly patients aged ≥65 years, whereas *M. pneumoniae* was more frequent in those aged <65 years. Antibiotic monotherapy was more frequent than combination therapy, and beta-lactams were the most commonly prescribed antibiotics. Hospitalised patients were more likely than outpatients to receive combination antibiotic therapy. Limited data on antibiotic resistance were available in the studies. Penicillin resistance of *S. pneumoniae* was reported in 8.4–20.7 % of isolates and erythromycin resistance was reported in 14.7–17.1 % of isolates. Understanding the aetiology of CAP and the changing pattern of antibiotic resistance in Europe, together with an increased awareness of the risk factors for CAP, will help clinicians to identify those patients most at risk of developing CAP and provide guidance on the most appropriate treatment.

**Electronic supplementary material:**

The online version of this article (doi:10.1007/s10096-014-2067-1) contains supplementary material, which is available to authorized users.

## Introduction

The clinical and economic burden of community-acquired pneumonia (CAP) in Europe is substantial. A review of the burden of CAP in Europe demonstrated that the incidence of CAP and hospitalisations for CAP are rising [1]. The incidence of CAP was shown to be higher in men than in women and to increase with age. In addition to increasing age [1–3], several other risk factors for CAP have been established, including smoking [2], immunosuppression [3] and the presence of comorbid conditions [4–7]. With an ageing population in Europe, the clinical and economic burden of CAP is expected to continue to rise over time, placing increasing pressure on hospital resources and society [1, 8–11].

*Streptococcus pneumoniae* is widely accepted as being the most common pathogen causing CAP. However, the frequency at which it is identified varies considerably between studies across Europe [1, 12]. In addition to *S. pneumoniae*, several other pathogens cause CAP, including atypical pathogens such as *Legionella pneumophila* and *Staphylococcus aureus*, and Gram-negative bacilli, including *Pseudomonas aeruginosa* [13–18].

It is important to understand the emerging role of different pathogens in the aetiology of CAP to effectively guide appropriate antibiotic management [19]. Inappropriate antibiotic treatment in patients with CAP has been repeatedly linked with worse outcomes [20–23]. This literature review was conducted to generate up-to-date information on the aetiology of CAP and its antibiotic management in adults across Europe.

## Methods

**Fig. 1:**
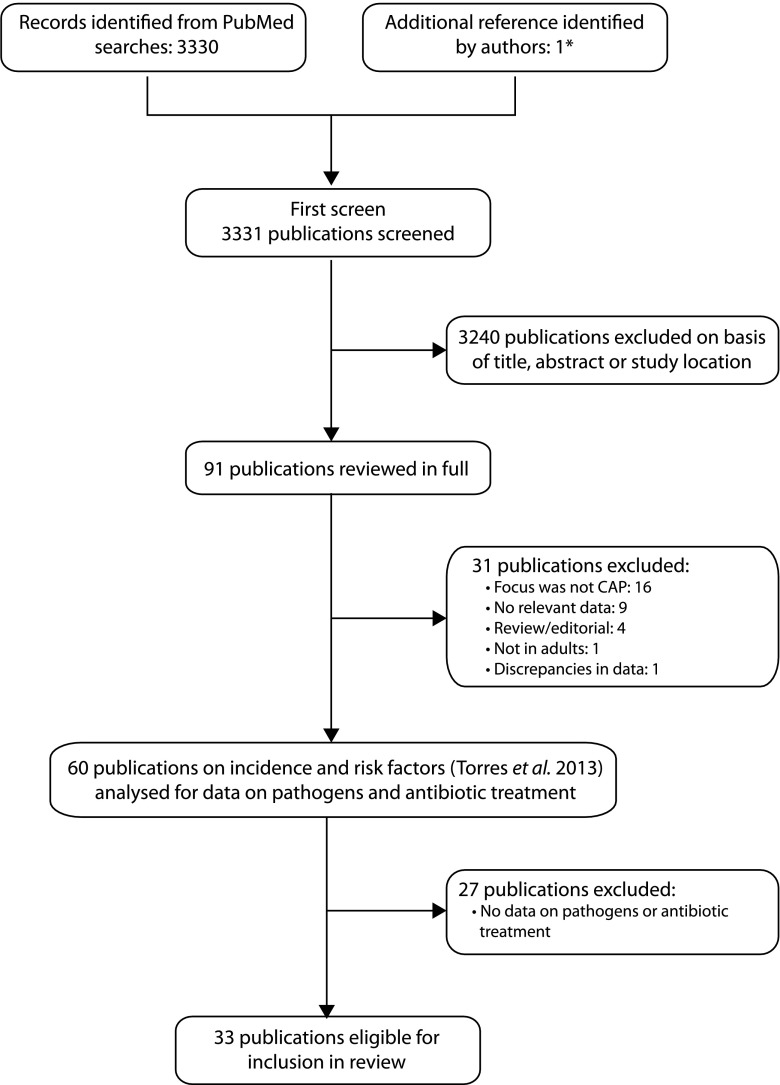
Summary of the study selection procedure. (Adapted from Fig. 1 of Torres et al. [7], used under the Creative Commons—Attribution-NonCommercial (CC BY-NC 3.0) license. The original can be found here: http://thorax.bmj.com/content/68/11/1057/F1.large.jpg). *CAP* community-acquired pneumonia. *One study did not include the terms ‘risk’ or ‘co-morbidity’/‘comorbidity’ in either the title or the abstract and, so, was not identified in the PubMed searches; however, ‘risk factors’ was included in the list of MeSH terms for the article

The search methodology for this literature review was the same as that described for a previous literature search and analysis [7], but with additional filters for the topics of interest to this review (Fig. 1).

The PubMed database was searched using the following search string: pneumonia AND English AND 2005/01/01–2012/07/31 AND risk NOT clinical trial, phase I OR clinical trial, phase II OR clinical trial, phase III OR controlled clinical trial OR randomized controlled trial OR case reports OR practice guideline OR editorial OR review OR cost OR cost effectiveness OR efficacy OR immunogenicity OR economic OR nosocomial. Additional searches used the same search string, but replaced ‘risk’ with either ‘comorbidity’ or ‘co-morbidity’.

Articles were included in the initial literature search [7] if they reported observational studies performed in Western European countries (Austria, Belgium, Denmark, Finland, France, Germany, Greece, Ireland, Italy, The Netherlands, Norway, Portugal, Spain, Sweden, Switzerland, UK) and presented data from individuals >15 years of age on either the incidence of CAP in at-risk individuals, defined as those with underlying risk factors placing them at increased risk of CAP (as defined in [7]), or risk factors for CAP. The papers identified were further screened for data on pathogens identified in patients with CAP and/or antibiotic treatment in patients with CAP. Studies that focused on nosocomial or healthcare-acquired pneumonia were excluded.

The included articles were reviewed in full and data on the study setting and methodology, characteristics of the populations studied, pathogens and antibiotic treatments were extracted. If more than one paper reported different aspects of the same study, all relevant papers were included. Where the same data were reported in more than one paper, the first paper to be published was selected for inclusion. The analysis of the included papers was descriptive and no meta-analyses of data were performed. Unless otherwise stated, all data are reported as odds ratios (ORs) (95 % confidence intervals [CIs]).

## Results

### Included studies

As reported previously [7], a total of 3,331 articles published between January 2005 and July 2012 were identified, of which 3,240 could be excluded on the basis of the title, abstract or study location. Further screening of the PubMed results and full papers identified 60 references meeting the inclusion and exclusion criteria. We summarise data from 33 of these studies that reported on the pathogens identified in patients with CAP and/or antibiotic treatment in patients with CAP. The included studies were performed in Denmark (*n* = 1), France (*n* = 3), Germany (*n* = 3), Greece (*n* = 1), Italy (*n* = 4), Spain (*n* = 20) and the UK (*n* = 1). Details of the study designs and populations are summarised in Table 1.

**Table 1:** Methodology and patient demographics of the studies included in the review. (Adapted from Supplementary Table 1 of Torres et al. [7], used under the Creative Commons—Attribution-NonCommercial (CC BY-NC 3.0) license. The original can be found here: http://thorax.bmj.com/content/suppl/2013/10/15/thoraxjnl-2013-204282.DC1/thoraxjnl-2013-204282supp_tables.pdf)

Citation	Country; region	Study method	Study period	Population	Age (yrs [mean ± SD])	Definition of CAP
Denmark						
Holm et al. 2007 [65]	Denmark; Odense	Multicentre, prospective, observational study	9 Sept–1 Nov 2002; 6 Jan–25 April 2003	Primary care patients ≥18 yrs with a diagnosis of community-acquired LRTI, *n* = 364; 48 with pneumonia	Overall, median 50 [range 18–94]	GP diagnosis of LRTI and chest X-ray confirmed
					Pneumonia, median 61 [range 22–88]	
France						
Le Moing et al. 2006 [35]	France; national	Multicentre, prospective, observational cohort study	May 1997–Dec 2001	HIV patients receiving protease inhibitor therapy, *n* = 1,203; 29 hospitalised with pneumonia	Median 36	Clinical symptoms, chest X-ray confirmed and microbiological data
Bénard et al. 2010 [33]	France; Aquitaine	Multicentre, prospective, cohort study	2000–2007	Patients with HIV, *n* = 3,336; 135 with bacterial pneumonia	Median 39.6 [IQR 34.5–46.0]	Chest X-ray confirmed and bacteriological identification or successful antibacterial treatment
Chidiac et al. 2012 [27]	France; metropolitan	Multicentre, prospective, observational, cohort study	1 April 2006–30 June 2007	Patients hospitalised with community-acquired Legionnaires’ disease, *n* = 540	60 [range 17–100]	Chest X-ray confirmed and laboratory evidence of *Legionella pneumophila* infection
Germany						
Klapdor et al. 2012 [41]	Germany; national (CAPNETZ)	Multicentre, prospective, observational study	Jan 2002–June 2009	Patients ≥18 yrs with CAP, *n* = 7,803; 4,083 <65 yrs (2.6 % nursing home residents); 3,720 ≥65 yrs (14.4 % nursing home residents)	Overall, 60.9 ± 18.5 [range 18–101]	Clinical symptoms, chest X-ray confirmed and microbiological data
					<65 yrs, median 47.0 [IQR 20.7]	
					≥65 yrs, median 76.0 [IQR 11.8]	
von Baum et al. 2010 [32]	Germany; national (CAPNETZ)	Multicentre, prospective, observational study	1 June 2002–30 June 2007	Patients with CAP, *n* = 5,130 (6 % nursing home residents); 67 with EB; 22 with PA; 1,833 with no definite EB/PA	Overall, 60 ± 18	Chest X-ray or clinical symptoms
					With EB, 73 ± 15	
					With PA, 64 ± 17	
					No EB/PA, 58 ± 18	
Kothe et al. 2008 [23]	Germany; national (CAPNETZ)	Multicentre, prospective, observational study	March 2003–Oct 2005	Patients with CAP, *n* = 2,647; 1,298 <65 yrs (3.3 % nursing home residents); 1,349 ≥65 yrs (15.2 % nursing home residents)	<65 yrs, 47.2 ± 12.7	Clinical symptoms, chest X-ray confirmed and microbiological data
					≥65 yrs, 77.1 ± 7.5	
Greece						
Kofteridis et al. 2009 [30]	Greece; Crete	Single-centre, retrospective, observational study	Jan 1996–Dec 2002	Adults hospitalised with community-acquired LRTI due to *Haemophilus influenzae n* = 45	Median 68 [range 28–86]	Clinical symptoms, chest X-ray confirmed and positive sputum culture for *H. influenzae*
Italy						
Madeddu et al. 2008 [36]	Italy; northern Sardinia	Single-centre, observational, retrospective analysis of consecutive patients	Jan 1999–Dec 2004	HIV patients hospitalised with CAP, *n* = 76; 84 episodes	38.3 ± 7.5 [range 27–80]	Clinical symptoms, chest X-ray confirmed and microbiological data
^a^Viegi et al. 2006 [47]	Italy, national	Multicentre, prospective, observational, population-based study	15 Feb 1999–14 Feb 2000	Primary care patients with CAP, *n* = 699; 548 diagnosed by GP (4.7 % nursing home residents); 151 diagnosed by hospital (8.9 % nursing home residents)	59.6 ± 19.5	Chest X-ray and clinical symptoms
					Patients diagnosed in community, 57.6 ± 19.2	
					Patients diagnosed in hospital, 66.7 ± 18.7	
^b^Manno et al. 2009 [37]	Italy; Brescia	Single-centre, prospective, observational, cohort study	June 2000–Dec 2006	HIV patients hospitalised with CAP	Cirrhosis, 41.0 ± 4.3	Clinical symptoms, chest X-ray confirmed and microbiological data
				Patients with cirrhosis, *n* = 29	No cirrhosis, 37.3 ± 6.2	
				Patients without cirrhosis, *n* = 73		
Migliorati et al. 2006 [66]	Italy; Brescia	Single-centre, observational, retrospective analysis	Jan 2001–Dec 2002	Patients ≥15 yrs hospitalised with discharge diagnosis of pneumonia or pneumonia-related disease, *n* = 148 (20 % nursing home residents)	70.3 ± 17.3	Chest X-ray confirmed
Spain						
Sopena et al. 2007 [28]	Spain; Barcelona	Single-centre, prospective, observational, cohort study	1994–2004	Adult patients hospitalised with community-acquired Legionnaires’ disease, *n* = 251; 138 sporadic cases; 113 outbreak cases	Sporadic cases, 56.6 ± 15.5	Laboratory evidence of acute infection with *L. pneumophila*
					Outbreak cases, 59.5 ± 16.6	
Sopena et al. 2007 [29]	Spain; Barcelona	Single-centre, retrospective, observational, cohort study	1994–2004	Patients hospitalised with CAP due to *L. pneumophila*, *n* = 158; 104 <65 yrs; 54 ≥65 yrs	<65, 65.9 % of cohort	Laboratory evidence of infection with *L. pneumophila*
					≥65, 34.1 %	
					≥70, 13.9 %	
					≥85, 1.9 %	
Garcia-Vidal et al. 2009 [48]	Spain; Barcelona	Single-centre, prospective, observational cohort study	1 Jan 1995–31 Dec 2005	Patients hospitalised with CAP, *n* = 1,556; 146 with recurrent CAP (≥2 episodes of CAP in 3 yrs with asymptomatic period ≥1 month); 1,410 with non-recurrent CAP	Recurrent CAP, 70.96 ± 13.824	Clinical symptoms, chest X-ray confirmed and microbiological data
					Non-recurrent CAP, 65.03 ± 16.573	
Falguera et al. 2009 [39]	Spain; Catalonia	Two-centre, prospective, observational cohort study	Jan 1995–Dec 2005	Patients ≥18 yrs hospitalised with CAP, *n* = 3,272; 61 with Gram-negative infections; 3,211 with non-Gram-negative infections	64 [range 18–100]	Clinical symptoms, chest X-ray confirmed and microbiological data
					Gram-negative infections, 69	
					Non-Gram-negative infections, 63	
Ruiz et al. 2010 [31]	Spain; Basque country	Single-centre, prospective, observational, cohort study	Jan 1995–Dec 2007	Adults hospitalised with bacteraemic CAP due to Gram-negative bacteria, *n* = 51	72.9 ± 11.3	Clinical symptoms, chest X-ray confirmed
Viasus et al. 2011 [46]	Spain; Barcelona	Single-centre, prospective, observational, cohort study	13 Feb 1995–31 Dec 2008	Patients with and without cirrhosis, hospitalised with CAP, *n* = 3,420; 90 with cirrhosis; 3,330 with no cirrhosis	Cirrhosis, 61.8 ± 13.0	Chest X-ray and clinical symptoms
					No cirrhosis, 66.8 ± 16.9	
Viasus et al. 2011 [50]	Spain; Barcelona	Single-centre, prospective, observational, cohort study	13 Feb 1995–30 April 2010	Adult patients with and without chronic renal disease, hospitalised with CAP, *n* = 3,800; 203 with renal disease (8.6 % nursing home residents); 3,597 with no renal disease (8.1 % nursing home residents)	Renal disease, median 77 [IQR 67–84]	Chest X-ray, clinical symptoms and microbiological data
					No renal disease, median 70 [IQR 56–79]	
de Roux et al. 2006 [45]	Spain; Barcelona	Single-centre, prospective, observational cohort study	Oct 1996–Nov 2001	Patients hospitalised with CAP, classified according to alcohol abuse status	Current alcohol abuse, 58 ± 14	Clinical symptoms, chest X-ray confirmed and microbiological data
				Current, *n* = 128	Former alcohol abuse, 71 ± 11	
				Former, *n* = 54	No alcohol abuse, 68 ± 19	
				None, *n* = 1,165		
Gutiérrez et al. 2005 [40]	Spain; Alicante	Single-centre, prospective, observational, cohort study	15 Oct 1999–14 Oct 2001	Patients ≥15 yrs with CAP, *n* = 493	56.6 [range 15–94]	Clinical symptoms, chest X-ray confirmed and microbiological data
Curran et al. 2008 [34]	Spain; Barcelona	Single-centre, prospective, observational cohort study	Jan 2000–Dec 2005	HIV patients ≥18 yrs hospitalised with bacterial pneumonia, *n* = 161; 186 episodes	39.7 ± 7.8	Clinical symptoms, chest X-ray confirmed and response to antibacterial therapy
Pérez-Sola et al. 2011 [67]	Spain; national	Multicentre, prospective, observational, cohort study	Feb 2000–Jan 2006	Patients with rheumatic diseases treated with TNF antagonists, *n* = 6,969; 101 with pneumonia	50 ± 14	CDC criteria
Carratalà et al. 2007 [43]	Spain; Barcelona	Single-centre, prospective, observational study	1 Jan 2001–31 Dec 2004	Adult patients with CAP requiring hospitalisation, *n* = 601	63.7 ± 17.1	Chest X-ray confirmed
Cabre et al. 2010 [25]	Spain; Mataró	Single-centre, prospective, observational study	Jan 2001–Aug 2005	Patients ≥70 yrs with CAP requiring hospitalisation, *n* = 134 (32 % nursing home residents)	84.51 ± 6.8	Chest X-ray confirmed
Cillóniz et al. 2012 [44]	Spain; Barcelona	Single-centre, prospective, observational cohort study	2001–2009	Adult patients hospitalised with pneumococcal pneumonia, *n* = 626	63.6 ± 18.9	Clinical symptoms, chest X-ray confirmed and microbiological data
					46 % ≤65 yrs	
Vila-Corcoles et al. 2009 [26]	Spain; Tarragona	Multicentre, prospective, observational, population-based, cohort study	1 Jan 2002–30 April 2005	Community-dwelling individuals ≥65 yrs, *n* = 11,241	65–74, 55.2 % of cohort	Chest X-ray and clinical symptoms
					75–84, 34.3 %	
					≥85, 10.5 %	
Cillóniz et al. 2011 [22]	Spain; Barcelona	Single-centre, prospective, observational cohort study	Jan 2003–Dec 2010	Patients with CAP admitted to ICU, *n* = 362	63.4 ± 16.5	Chest X-ray confirmed
Molinos et al. 2009 [38]	Spain; Asturias	Multicentre, prospective, observational study	April 2003–April 2004	Patients hospitalised with CAP, *n* = 710; 244 with COPD; 466 no COPD (5 % nursing home residents in both groups)	Overall, 67.14 [95 % CI 65.9–68.4]	Clinical symptoms, chest X-ray confirmed and microbiological data
					With COPD, 73.7 [95 % CI 72.5–74.9]	
					No COPD, 63.6 [95 % CI 61.8–65.4]	
Liapikou et al. 2012 [42]	Spain; Barcelona	Single-centre, prospective, observational cohort study	2004–2008	Adult patients hospitalised with CAP, *n* = 1,379; 212 with COPD (5.7 % nursing home residents); 1,167 no COPD (10.2 % nursing home residents)	Overall, 70 ± 17	Clinical symptoms, chest X-ray confirmed and microbiological data
					COPD, 73.4 ± 8.8	
					No COPD, 69.4 ± 17.9	
Almirall et al. 2013 [24]	Spain; Mataró	Single-centre, prospective, observational, case–control study	Feb 2008–Feb 2010	Patients ≥70 yrs with CAP requiring hospitalisation	Cases, mean ± SEM 81.22 ± 0.77	Chest X-ray confirmed and bacteriological identification
				Cases, *n* = 36	Controls, mean ± SEM 81.21 ± 0.53	
				Controls, *n* = 72		
Giannella et al. 2012 [49]	Spain; national	Multicentre, prospective, observational cohort study	Jan and June 2010 (1 week in each month)	Patients ≥16 yrs treated for CAP in the internal medicine department, *n* = 591	Median 77 [IQR 65–84]	Clinical symptoms, chest X-ray confirmed
UK						
Bewick et al. 2012 [68]	UK; Nottingham	Two-centre, prospective, observational cohort study	Sept 2008–Sept 2010	Patients ≥16 yrs hospitalised with CAP, *n* = 920 (5.5 % nursing home residents); 366 with pneumococcal pneumonia (6.8 % nursing home residents)	Median 71.7 [IQR 57.8–80.8]	Chest X-ray confirmed

*CAP* community-acquired pneumonia; *CAPNETZ* Competence Network for Community-Acquired Pneumonia; *CDC* Centers for Disease Control and Prevention; *COPD* chronic obstructive pulmonary disease; *EB* Enterobacteriaceae; *GP* general practitioner; *HIV* human immunodeficiency virus; *ICU* intensive care unit; *IQR* interquartile range; *LRTI* lower respiratory tract infection; *PA Pseudomonas aeruginosa*; *SD* standard deviation; *SEM* standard error of the mean; *TNF* tumour necrosis factor; *yrs* years

^a^Ten patients in this study were aged ≤14 yrs

^b^This paper refers to patients with cirrhosis as ‘Cases’ and those without cirrhosis as ‘Controls’. However, there is no evidence of any matching of ‘cases’ and ‘controls’

The majority of studies included adults of all ages, but three studies considered only elderly patients (age ≥65 years) [24–26]. Additionally, most of the studies considered pneumonia of any aetiology, but six were performed in patients with pneumonia due to *L. pneumophila* (*n* = 3) [27–29], *Haemophilus influenzae* (*n* = 1) [30], Gram-negative bacteria (*n* = 1) [31], or *Enterobacteriaceae* or *P. aeruginosa* (*n* = 1) [32]. Six studies were conducted in specific populations: five studies in patients with human immunodeficiency virus (HIV) [33–37] and one study in patients with chronic obstructive pulmonary disease (COPD) [38].

### Pathogens identified in patients with CAP

The aetiology of CAP was investigated in 25 observational studies in Denmark (*n* = 1), France (*n* = 2), Germany (*n* = 2), Italy (*n* = 2), Spain (*n* = 17) and the UK (*n* = 1). Tables 2, 3 and 4 summarise these data to show the most common microbiological techniques, the overall frequency of isolation of pathogens and the frequency of isolation of pathogens specifically in HIV and COPD study cohorts. Full details of the microbiological techniques used and the pathogens isolated in each study are included in Supplementary Table 1.

**Table 2:** Microbiological techniques/samples used for the isolation of pathogens in patients with CAP

Microbiological technique/sample	Number of studies using technique, *n* (%)	References
Blood culture	22 (100)	[22–24, 26, 31, 34–43, 45, 46, 48–50, 65, 68]
Sputum culture	20 (91)	[22–24, 26, 34, 36–43, 45, 46, 48–50, 65, 68]
Urine antigen test^a^	19 (86)	[22–24, 26, 31, 34, 36, 38–43, 45, 46, 48–50, 68]
Blood serology^b^	15 (68)	[22, 26, 36, 38–43, 45, 46, 48–50, 68]
Pleural fluid	10 (45)	[22–24, 34, 38–40, 42, 45, 50]
Tracheobronchial aspirate	7 (32)	[22–24, 38, 42, 45, 49]
Bronchoalveolar lavage	6 (27)	[22, 23, 37, 45, 49, 68]
Transthoracic needle aspirate	4 (18)	[23, 39, 45, 49]
Normally sterile fluid culture	3 (14)	[43, 46, 48]
Nasopharyngeal swab	3 (14)	[22, 41, 49]
Sublingual smear	1 (5)	[24]

*CAP* community-acquired pneumonia; *n* number of studies using the given technique of the 22 studies reporting the microbiological techniques used for the isolation of pathogens in patients with CAP

^a^For the detection of *Streptococcus pneumoniae* and *Legionella pneumophila*

^b^For the detection of antibodies against specific pathogens or groups of pathogens, including *Legionella pneumophila*, *Chlamydophila pneumoniae*, *Coxiella burnetii*, *Mycoplasma pneumoniae*, *Chlamydophila psittaci*, *Chlamydia trachomatis* and respiratory viruses

**Table 3:** Patients with CAP and episodes of CAP with a pathogen identified

Aetiology	Patients with pathogen identified^a^			Episodes with pathogen identified^a^			References
	Cohorts (*n*)^b^	Studies (*n*)	Range (%)	Cohorts (*n*)^b^	Studies (*n*)	Range (%)	
Gram-positive bacteria							
*Streptococcus pneumoniae*	51	19	12.0–85.0	6	5	3.2–19.2	[22–26, 33–43, 45, 46, 48–50, 65, 67, 68]
*Staphylococcus aureus*	39	12	0.8–20.0	2	2	3.3–6.5	[22, 23, 26, 33, 36, 38, 40–42, 46, 49, 50, 65, 67]
*Streptococcus viridans*	1	1	1.7	1	1	3.3	[22, 36]
Gram-negative bacteria							
Gram-negative enteric bacilli^c^	39	10	0.6–42.9	3	2	1.7–7.8	[22, 23, 25, 34, 37, 40, 41, 45, 46, 48–50]
*Haemophilus influenzae*	45	15	1.1–29.4	6	5	3.2–19.2	[22, 23, 25, 26, 33–43, 45, 46, 48–50, 65]
*Pseudomonas aeruginosa*	14	10	0.9–16.8	2	2	5.9–6.7	[22, 24, 26, 34, 36, 38, 41–43, 45, 49, 67]
*Pseudomonas* species^d^	19	1	0.2–3.2	1	1	19.4	[33, 41]
*Klebsiella pneumoniae*	5	5	0.3–5.0	1	1	3.3	[24, 26, 36, 38, 42, 43]
*Moraxella catarrhalis*	28	5	0.3–2.3	0	0	–	[26, 40–42, 46]
*Serratia marcescens*	1	1	2.3	1	1	3.3	[26, 36]
*Escherichia coli*	5	3	0.6–2.1	1	1	6.7	[36, 38, 42, 43]
Atypical bacteria							
*Mycoplasma pneumoniae*	39	10	0.7–61.3	0	0	–	[22, 23, 38–43, 45, 65]
*Legionella pneumophila*	19	12	1.7–20.1	5	4	3.2–15.1	[22, 25, 26, 34–36, 38–40, 42, 43, 45, 46, 48–50]
*Legionella* species^e^	27	3	5.4–20.0	0	0	–	[23, 41, 67]
*Chlamydophila pneumoniae*	29	9	0.1–9.9	0	0	–	[22, 23, 26, 38, 39, 41–43, 45]
*Coxiella burnetii*	9	6	0.8–3.4	0	0	–	[22, 26, 38, 40, 43, 45]
Virus	38	10	1.4–28.6	1	1	0.7	[22, 23, 38, 40–42, 45, 46, 48, 49, 65]

*CAP* community-acquired pneumonia

Pathogens only reported in one cohort in one study were excluded

^a^Percentages are based on the number of patients/episodes in which pathogens were identified and data were available

^b^For studies that only reported data separately for each cohort, all cohorts were included; for studies that reported data for the overall study population, the summary data were used. Studies performed in patients with pneumonia due to a specific pathogen were excluded

^c^For studies [22, 23, 25, 34, 37, 40, 41, 45, 46, 48–50], Gram-negative enteric bacilli were grouped together and individual pathogens in this group were not reported separately

^d^For studies [33, 41], *Pseudomonas* species were not reported separately and, therefore, could include *P. aeruginosa*

^e^For studies [23, 41, 67], *Legionella* species were not reported separately and, therefore, could include *L. pneumophila*

**Table 4:** Prevalence of pathogens identified in patients with CAP with HIV or COPD

Aetiology	HIV			COPD		
	Patients with pathogen identified^a^	Episodes with pathogen identified^a^	References	Patients with pathogen identified^a^		References
	Range (%)	Range (%)		Range (%)		
				COPD	No COPD	
Gram-positive bacteria						
*Streptococcus pneumoniae*	57.8–81.8	42.9–71.4	[33–37]	37.5–66.3	26.9–57.0	[38, 40, 42]
*Staphylococcus aureus*	6.5	3.3	[33, 36]	1.1	0.8–3.2	[38, 40, 42]
Gram-negative bacteria						
Gram-negative enteric bacilli^b^	7.8	7.1–42.9	[33, 34]	16.7	3.1	[40]
*Haemophilus influenzae*	3.2–9.1	6.7–14.3	[33–37]	1.1–4.2	1.7–3.8	[38, 40, 42]
*Pseudomonas aeruginosa*	5.9	6.7	[34, 36]	2.1–7.4	0.9	[38, 42]
*Escherichia coli*	–	6.7	[36]	1.1–2.6	1.1–1.3	[38, 42]
*Klebsiella pneumoniae*	–	3.3	[36]	1.1	0.9	[38, 42]
*Moraxella catarrhalis*	–	–		2.1	0.4	[40, 42]
*Mycoplasma pneumoniae*	–	–		2.1–4.2	3.4–23.1	[38, 40, 42]
*Legionella pneumophila*	9.1–10.8	3.3	[34–36]	2.1–12.5	1.7–3.8	[38, 40, 42]
*Chlamydophila pneumoniae*	–	–		2.1–6.3	4.1–4.5	[38, 40, 42]
*Coxiella burnetii*	–	–		2.1	1.5–3.4	[38, 40]
Virus	–	–		4.2–13.7	2.8–12.5	[38, 40, 42]

*CAP* community-acquired pneumonia; *COPD* chronic obstructive pulmonary disease; *HIV*, human immunodeficiency virus

^a^Percentages are based on the number of patients/episodes in which pathogens were identified and data were available

^b^For studies [33, 34, 40], Gram-negative enteric bacilli were grouped together and individual pathogens in this group were not reported separately

Microbiological methodologies used to establish the aetiology of CAP were reported in 67 % of the studies (*n* = 22). These methodologies were similar across studies and included the assessment of blood, sputum, urine and pleural fluid samples and, less commonly, tracheobronchial, bronchoalveolar, transthoracic and nasopharyngeal samples. Blood cultures were performed in all 22 studies (Table 2) and all but one study reported using at least two different techniques. Other frequently used techniques for the isolation of pathogens were sputum culture (91 % of studies), urine antigen tests (specifically for the detection of *S. pneumoniae* and *L. pneumophila*; 86 % of studies), serology, for the detection of antibodies against specific pathogens, including *L. pneumophila*, *Chlamydophila pneumoniae*, *Coxiella burnetii*, *Mycoplasma pneumoniae*, *Chlamydophila psittaci*, *Chlamydia trachomatis* and respiratory viruses (68 % of studies), and pleural fluid culture (45 % of studies).

The percentages of patients and episodes of CAP in which a pathogen was not identified were 26.7–87.3 % and 44.2–77.0 %, respectively. In patients in whom a pathogen was identified, *S. pneumoniae* was the most commonly isolated and was identified in 12.0–85.0 % of patients within 19 studies (Table 3). Of the atypical bacteria, *M. pneumoniae* (up to 61.3 % of patients within ten studies), *L. pneumophila* (up to 20.1 % of patients within 12 studies) and *C. pneumoniae* (up to 9.9 % of patients within nine studies) were frequently identified in patients with CAP, whereas *C. burnetii* was isolated less frequently (up to 3.4 % of patients within six studies). Other pathogens isolated included *S. aureus* (up to 20.0 % of patients within 12 studies), *P. aeruginosa* (up to 16.8 % of patients within ten studies), *Klebsiella pneumoniae* (up to 5.0 % of patients within five studies) and *Acinetobacter baumannii* (isolated in 2.0 % of patients in one study that was performed in patients hospitalised with bacteraemic CAP due to Gram-negative bacteria [31]). CAP of mixed aetiology was reported in four studies in 0.4–19.9 % of patients [22, 26, 39, 40].

For studies with data available stratified by age (<65 years and/or ≥65 years) [23–26, 40, 41], the frequencies of pathogens were generally similar between age groups. However, *S. pneumoniae* (<65 years: 20.9–28.0 %; ≥65 years: 19.9–85.0 %), *H. influenzae* (<65 years: 4.1–6.4 %; ≥65 years: 2.9–29.4 %) and respiratory viruses (<65 years: 4.6–7.7 %; ≥65 years: 7.8–18.6 %) appeared to be more frequently isolated in elderly patients aged ≥65 years, and *M. pneumoniae* appeared to be more frequently isolated in younger patients (<65 years: 14.0–25.1 %; ≥65 years: 0.7–6.8 %).

Among the studies reporting on the aetiology of CAP in patients with HIV [33–37], the frequencies of isolated pathogens were similar to those found for the overall data. *S. pneumoniae* was the most commonly isolated pathogen (57.8–81.8 % of patients), and *H. influenzae* and *L. pneumophila* were also often identified (Table 4).

The aetiology of CAP was similar in patients with and without COPD [38, 40, 42], in whom *S. pneumoniae*, *H. influenzae*, *L. pneumophila*, *M. pneumoniae* and respiratory viruses were all commonly identified (Table 4). In one study, *P. aeruginosa* was reported in a significantly higher percentage of patients with COPD than in those without COPD (7.4 % vs. 0.9 %; *p* < 0.01) and *L. pneumophila* was found to be significantly lower in patients with COPD than in those without COPD (2.1 % vs. 7.8 %; *p* < 0.05) [42].

### Antibiotic treatment in patients with CAP

The antibiotic treatment of patients with CAP was reported in 23 studies: France (*n* = 1), Germany (*n* = 3), Greece (*n* = 1), Italy (*n* = 4) and Spain (*n* = 14). Rates of antibiotic treatment with beta-lactams, macrolides and quinolones are summarised in Table 5. Full details of the antibiotic therapies for each study are provided in Supplementary Table 2.

**Table 5:** Antibiotic treatment in adults with CAP

Antibiotic	Cohorts (*n*)^a^	Studies (*n*)	Patients treated with antibiotic^b^, range (%)	References
Monotherapy	31	7	16.0–94.7	[22, 40, 41, 43, 44, 46, 47]
Beta-lactams	32	8	5.0–87.7	[22, 37, 38, 40, 41, 43, 44, 47]
Macrolides	30	6	0.3–47.7	[37, 38, 40, 41, 44, 47]
Quinolones	32	8	2.0–46.0	[22, 37, 38, 40, 41, 43, 44, 47]
Other	26	3	0.7–8.8	[40, 41, 43]
Combination therapy	33	8	5.0–84.0	[22, 37, 40, 41, 43, 44, 46, 47]
Beta-lactam + macrolide	10	7	1.7–70.0	[22, 38, 40, 43–45, 47]
Beta-lactam + quinolone	4	4	6.3–63.0	[22, 43, 44, 47]
Macrolide + quinolone	2	2	0.9–1.0	[44, 47]
Other	5	4	2.0–38.0	[38, 43, 44, 47]

*CAP* community-acquired pneumonia

^a^For studies that only reported data separately for each cohort, all cohorts were included; for studies that reported data for the overall study population, the summary data were used. Studies performed in patients with pneumonia due to a specific pathogen were excluded

^b^Percentages are based on patients with available data

The rates of antibiotic treatments in patients with CAP were available in 13 studies [22, 28, 29, 36–38, 40, 41, 43–47]. The rate of monotherapy ranged from 16.0 to 94.7 % of patients and the rate of combination antibiotic therapy ranged from 5.0 to 84.0 % of patients (Table 5). The rate of antibiotic monotherapy with beta-lactams was higher than that for macrolides and quinolones. In one study, younger patients (<65 years) received fewer beta-lactams and more quinolones than older patients (≥65 years) (beta-lactams: 62.5 % vs. 81.3 %; quinolones: 28.2 % vs. 17.1 %, respectively), whereas macrolide use was similar between age groups (32.6 % vs. 31.4 %, respectively) [41]. For combination therapy, the most common combinations were beta-lactams combined with macrolides or quinolones. We found that the rate of combination antibiotic therapy was higher in patients in an intensive care unit (ICU; 84.0 %) and other hospitalised patients (31.8–69.0 %) than in outpatients (5.0–29.9 %) (Table 6). Three studies reported data on antibiotic treatment in populations with comorbidities (COPD [38] and liver disease [46]) or lifestyle risk factors for CAP (alcoholism [45]). Antibiotic treatments did not differ according to the presence or absence of COPD, liver disease or alcoholism.

**Table 6:** Antibiotic treatment in adults with CAP stratified by ICU patients, hospitalised patients and outpatients

Antibiotic	ICU patients		Hospitalised patients		Outpatients		References
	Cohorts (*n*)^a^	Patients treated with antibiotic^b^, range (%)	Cohorts (*n*)^a^	Patients treated with antibiotic^b^, range (%)	Cohorts (*n*)^a^	Patients treated with antibiotic^b^, range (%)	
Monotherapy	1	16.0	12	30.3–68.2	9	70.1–94.7	[22, 41, 43–47]
Beta-lactams	1	5.0	13	8.0–87.7	9	40.1–48.9	[22, 27, 37, 38, 41, 43, 45, 47]
Macrolides	–	–	12	0.3–47.7	9	14.1–22.1	[22, 27, 37, 38, 41, 45, 47]
Quinolones	1	11.0	13	2.0–46.0	9	12.0–39.2	[22, 37, 38, 41, 43–45, 47]
Other	–	–	9	0.7–3.6	8	5.1–8.8	[41, 43, 45]
Combination therapy	1	84.0	14	31.8–69.0	9	5.0–29.9	[22, 37, 41, 43–47]
Beta-lactam + macrolide	1	21.0	7	1.7–70.0	1	0.9	[22, 38, 43–47]
Beta-lactam + quinolone	1	63.0	2	27.0–28.1	1	6.3	[22, 43, 44, 47]
Macrolide + quinolone	–	–	1	1.0	1	0.9	[44, 47]
Other	–	–	4	2.0–38.0	1	11.4	[38, 43, 44, 47]

*CAP* community-acquired pneumonia; *ICU* intensive care unit

^a^For studies that only reported data separately for each cohort, all cohorts were included; for studies that reported data for the overall study population, the summary data were used. Studies performed in patients with pneumonia due to a specific pathogen were excluded

^b^Percentages are based on patients with available data

In the 14 studies that reported on appropriate versus inappropriate antibiotic therapy [22, 23, 27–31, 43, 44, 46–50], the majority of patients had received adequate initial antibiotic treatment. Inappropriate antibiotic therapy was reported in 0–39.0 % of patients [22, 23, 27–32, 43, 44, 46, 48–50]. One study showed that patients with polymicrobial CAP were significantly more likely than those with monomicrobial CAP (*p* < 0.001) to receive inappropriate antibiotic treatment (39.0 % vs. 10.0 %, respectively) [22]. Furthermore, inappropriate antibiotic therapy was found to be an independent predictor of mortality (univariate analysis: OR 11.23 [95 % CI 4.44–28.38], *p* < 0.001; multivariate analysis: adjusted OR 10.79 [3.97–29.30], *p* < 0.001) in one study [22].

The antibiotic resistance of pathogens responsible for CAP was described in four studies conducted in 1995–2008 [46], 2001 [45], 2001–2004 [43], 2001–2009 [44] and 2002 [30]. Penicillin resistance of *S. pneumoniae* was reported in 14.9–25.7 % of patients with CAP and in 8.4–20.7 % of isolates. Erythromycin resistance of *S. pneumoniae* was observed in 12.0–21 % of patients with CAP and in 14.7–17.1 % of isolates. In the two studies reporting on the antibiotic resistance of *H. influenzae*, beta-lactamase production was reported in 9.7 % [43] and 80.0 % [30] of isolates.

## Discussion

This review provides a comprehensive overview of the aetiology of CAP and its antibiotic treatment in patients in Western Europe and builds on knowledge from earlier reviews of the incidence and risk factors for CAP among adults in this region [7], the burden of CAP in Europe [1] and a meta-analysis on the incidence of CAP in Europe by Rozenbaum et al. [12]. In addition, it provides important information to be taken into consideration in future updates to the European guidelines for the management of CAP.

### Microbiological methodologies for the isolation of pathogens

The majority of studies were in patients hospitalised for CAP and, as may be expected, blood cultures were used for the isolation of pathogens. This is in line with current guidelines from the European Respiratory Society (ERS) and the European Society for Clinical Microbiology and Infectious Diseases (ESCMID) for the management of lower respiratory tract infections, which recommend that two sets of blood cultures are performed in patients hospitalised for CAP [18]. However, a study conducted between 2007 and 2011 in 14 countries in Europe found that blood cultures were performed in only 50 % of patients hospitalised with CAP [51]. This is similar to findings from a further retrospective, observational study in Europe conducted between 2010 and 2011 and published after the cut-off date for our search, in which blood cultures were performed in 55 % of patients hospitalised with CAP, suggesting that implementation of the guidelines across Europe is still incomplete [52]. It is also possible that some European hospitals have adopted the approach advocated within the most recent Infectious Diseases Society of America (IDSA)/American Thoracic Society (ATS) recommendations, which limit blood cultures to patients hospitalised in the ICU [53].

It is important to note that there are limitations in the methodologies used to evaluate the aetiology of CAP; for example, it is difficult to obtain all types of samples in all patients and many patients have received antibiotic treatment prior to sampling. Further limitations include the difficulty in obtaining a reliable sputum sample in the early stages of CAP in non-COPD patients and the technical limitations of diagnostic tests, such as a lack of sensitivity, or the poor ability of patients to form antibodies. Such limitations can lead to inaccurate estimations of the prevalence of pathogens [54]. Furthermore, there are often a substantial number of patients in which the aetiology of CAP cannot be identified. Therefore, the rates of isolated pathogens reported in studies could be under- or overestimated due to false-negative or false-positive results, or the inability to isolate a pathogen [54].

### Aetiology of CAP in Europe

We found that *S. pneumoniae* was the most common pathogen isolated in patients with CAP in Europe across the studies included in our review. However, there was substantial variation in the incidence of this pathogen (12.0–85.0 % of patients), which is comparable with findings from Welte et al., which identified *S. pneumoniae* in 11.9–68.3 % of patients with CAP, and from the meta-analysis by Rozenbaum et al. on the incidence of CAP in Europe, which identified *S. pneumoniae* in 19.3 % of CAP episodes [1, 12].

Other frequently identified pathogens found to cause CAP across the included studies in our review were *H. influenzae*, Gram-negative enteric bacilli, respiratory viruses and *M. pneumoniae*. These pathogens were identified at rates similar to those found by Welte et al. [1], with the exception of *M. pneumoniae*, which was higher in our review (61.3 % vs. 32.4 % of patients). The high level of *M. pneumoniae* in our review was influenced by one study in particular, which consistently identified high *M. pneumoniae* rates in the age cohorts studied, particularly the younger cohorts [41]. A possible reason for these high rates of *M. pneumoniae* is the cyclical nature of *M. pneumoniae* outbreaks, which occur every 3–7 years; hence, the prevalence of this organism in a given study varies with the inclusion of such yearly epidemics. Secondly, the study methodology for isolating *M. pneumoniae* used polymerase chain reaction (PCR) from bronchoalveolar lavage and throat swab samples, which were available for almost all patients, as well as sputum samples, which were available in only approximately 40 % of patients. Therefore, the true denominator for calculating *M. pneumoniae* rates differed from that for other pathogens, which resulted in an overestimation of the *M. pneumoniae* rate by two-fold [41].

We found that the multidrug-resistant pathogens accounted for ≤20.0 % of CAP and that, of these pathogens, *S. aureus* and *P. aeruginosa* were more frequently isolated than *K. pneumoniae* or *A. baumannii*, which were rarely identified as the cause of CAP. In a European study of pathogens in hospitalised patients with CAP, multidrug-resistant pathogens were the cause of CAP in 3.3–7.6 % of patients in which a pathogen could be identified, with methicillin-resistant *S. aureus* being the most common multidrug-resistant pathogen [55]. The study also found that patients with CAP caused by multidrug-resistant pathogens typically presented with more severe pneumonia on admission to hospital and, correspondingly, multidrug-resistant pathogens were more prevalent among those patients admitted to an ICU than among those admitted to a general ward [55]. Overall, multidrug-resistant pathogens do not appear to be a major cause of CAP in Europe, but the severity of CAP caused by multidrug-resistant pathogens highlights the importance of routine testing for these pathogens. Probabilistic scores, such as the Aliberti and Shorr scores, can be useful for predicting the presence of multidrug-resistant pathogens in hospitalised patients and could help physicians to prescribe appropriate treatments without overprescribing broad-spectrum antibiotics [55].

The frequency of pneumonia of mixed aetiology varied across the four studies in which it was identified, from 0.4 to 19.9 % of patients. One important factor likely to contribute to this variation is the diagnostic methods used to identify pathogens. It is probable that a higher percentage of polymicrobial infections will be identified using newer molecular techniques. These techniques are also likely to contribute to an increase in the percentage of infections in which a causative pathogen can be identified, reducing underdiagnosis and increasing the accuracy of diagnoses, which will potentially lead to improvements in the accuracy of treatment.

Differences in the groupings of pathogens between studies (e.g. Gram-negative enteric bacilli ± *P. aeruginosa*) may have led to underestimations in the prevalence of some pathogens, such as *P. aeruginosa*. As for *S. pneumoniae*, we generally found large ranges in the frequency of pathogens isolated across studies. Differences in the isolation rates of pathogens between studies could be due to many factors, including the severity of CAP, healthcare settings (e.g. patients treated in the community versus in the ICU), populations studied (e.g. age, comorbidities, risk factors) and diagnostic tests used (e.g. traditional methods versus new technology). For example, in a meta-analysis of the prevalence of *S. pneumoniae* in Europe, *S. pneumoniae* was more likely to be detected in studies that used PCR assays compared with studies that used other diagnostics tests (OR 2.49 [95 % CI 1.39–4.46]) [12]. To establish the aetiology of CAP in Europe more accurately, improvements are needed in the sensitivity and specificity of diagnostic tests used to isolate pathogens. Furthermore, a more standardised approach to the diagnostic tests used will make comparisons across different studies more valid.

When looking at the aetiology of CAP stratified by age, we found a trend for *S. pneumoniae*, *H. influenzae* and respiratory viruses to be more frequent in elderly patients aged ≥65 years, and *M. pneumoniae* to be more frequent in those aged <65 years. Similar age-related trends have been observed previously in a study of the microbial aetiology of CAP in adults in Finland [54], in which *S. pneumoniae* infections were more frequent in adults aged ≥60 years than in those aged <60 years (48 % vs. 35 %, *p* = 0.04) and infections with *M. pneumoniae* were more frequent in individuals aged 15–44 years compared with older adults (24 % vs. 3 %, *p* < 0.001). The study in Finland also found viruses to be the cause of CAP in a higher proportion of older adults than younger adults; however, this trend was not significant. No consistent age-related trend was observed for *H. influenzae* [54]. A study of the microbial patterns of CAP in patients aged ≥65 years found that *S. pneumoniae* was the most frequent pathogen in all age groups over 65 years and that age did not influence the microbial cause of CAP [6]. In patients with COPD, we found that the aetiology of CAP was similar to that in patients without this condition. This was also observed in patients with HIV (in those who were non-severely immunocompromised or receiving treatment), which is in line with the results from studies examining the impact of HIV on the clinical outcomes of CAP in the highly active antiretroviral therapy era. Non-severely immunocompromised patients with HIV have been shown to have similar clinical outcomes in terms of the time to clinical stability, length of hospital stay and mortality rate when compared with individuals without HIV [56, 57].

### Antibiotic treatment in patients with CAP

Current ERS/ESCMID guidelines (2011 edition) for the treatment of CAP [18] recommend one of the following for the treatment of CAP in hospitalised patients:

- Aminopenicillin ± macrolide
- Aminopenicillin beta-lactamase inhibitor ± macrolide
- Non-antipseudomonal cephalosporin III
- Cefotaxime or ceftriaxone ± macrolide
- Levofloxacin
- Moxifloxacin
- Penicillin G ± macrolide

The ERS/ESCMID guidelines suggest that combination therapy should be restricted to patients with severe presentation of CAP, with combination therapy being the treatment of choice for patients with severe CAP being treated in the ICU [18]. In these patients, non-antipseudomonal cephalosporin III plus a macrolide, or moxifloxacin or levofloxacin ± non-antipseudomonal cephalosporin III are recommended in those patients without risk factors for *P. aeruginosa*, whereas in patients with risk factors for *P. aeruginosa*, antipseudomonal cephalosporin, or acylureidopenicillin beta-lactamase inhibitor or carbapenem, plus ciprofloxacin or plus macrolide plus aminoglycoside is preferred. When a specific pathogen has been identified, antibiotic therapy can be targeted against that pathogen. For example, for CAP caused by *Legionella* species, respiratory quinolones are recommended [18]. In our study, we found that beta-lactams and macrolides were more frequently prescribed than quinolones. This was not unexpected, as some treatment guidelines reserve quinolones for when initial empirical therapy has failed, or specifically for the treatment of CAP caused by *Legionella* species (which was identified in up to 20.1 % of patients in this review) [58].

Many European countries have their own national guidelines for the treatment of CAP [54, 58–63], which are derived from the European guidelines and take into account the local epidemiology and aetiology of CAP, as well as the national resistance rate against antibiotics, such as penicillins and macrolides. This means that, although some similarities exist and the European guidelines provide a good framework for guidance, there are variations in the antibiotic management of CAP throughout Europe, depending on the specific requirements of each country.

A limited amount of data regarding antibiotic resistance were reported in the studies included in this review. By contrast, Welte et al. found several studies with data on antibiotic resistance [1], possibly due to the use of different literature search criteria (e.g. differences in the time periods reviewed, databases searched, search terms used and inclusion/exclusion criteria applied). Antibiotic resistance against *S. pneumoniae* is the main clinical concern, due to its dominance in the aetiology of CAP. We found that pneumococcal resistance against penicillin was slightly higher than might be expected (8.4–20.7 % of isolates [44, 46]) when compared with that reported in Europe by the European Antimicrobial Resistance Surveillance Network (EARS-Net) in 2011 (8.8 % of isolates non-susceptible and 2.3 % resistant) [64]. However, this may be because all of the studies in this review reporting penicillin resistance were carried out in Spain, which has one of the highest levels of penicillin resistance of *S. pneumoniae* in Europe [64]. Pneumococcal resistance against erythromycin was similar to that reported by the EARS-Net for macrolides (14.7–17.1 % of isolates [43–46] vs. 14.1 % of isolates) [64]. The literature review by Welte et al. highlighted a trend for increased antibiotic resistance of CAP-related pathogens in Europe, including *S. pneumoniae*, which showed an increase in resistance to commonly prescribed antibiotics [1]. Globalisation and developments in healthcare may contribute to the changing pattern of the aetiology and antibiotic resistance of CAP. Understanding these changes is essential to guide best practices in the antibiotic management of CAP and to safeguard against the failure of empiric antibiotic treatment. The implementation of global surveillance systems would provide a means for guidelines to be adapted more rapidly in response to such changes.

### Strengths and limitations

This literature review was based on a review of published data from Europe that aimed to capture as many studies as possible from the past 7 years. The main strength of this review is that many of the included publications were case–control studies performed with large numbers of patients drawn from registries or primary care databases, rather than small, single-centre studies, thus giving reassurance that they provide a good representation of CAP in European populations. However, this review also has some limitations. There was a lack of a well-defined diagnostic protocol in many of the studies and the percentage of patients or episodes of CAP in which a specific causative pathogen was not identified was high in some studies (26.7–87.3 % and 44.2–77.0 %, respectively). The majority of the included studies were based on patient populations in Spain (20 of 33 studies) and this could potentially limit the validity of extrapolating the data from this review to other European populations.

## Conclusion

In conclusion, this review has highlighted that *Streptococcus pneumoniae* is the most common pathogen responsible for community-acquired pneumonia (CAP) in adults in Europe and that beta-lactams are the most frequently prescribed class of antibiotics for the treatment of CAP. Understanding the aetiology of CAP and the changing pattern of antibiotic resistance in Europe, together with an increased awareness of the risk factors for CAP, will help clinicians to identify those patients most at risk of developing CAP and provide guidance on the most appropriate treatment.

## Electronic supplementary material

Below are the links to the electronic supplementary material.

Supplementary Table 1 (DOC 0.98 mb)

Supplementary Table 2 (DOC 443 kb)

## References

[1] Welte T, Torres A, Nathwani D (2012). Clinical and economic burden of community-acquired pneumonia among adults in Europe. Thorax.

[2] Baik I, Curhan GC, Rimm EB, Bendich A, Willett WC, Fawzi WW (2000). A prospective study of age and lifestyle factors in relation to community-acquired pneumonia in US men and women. Arch Intern Med.

[3] Koivula I, Sten M, Mäkelä PH (1994). Risk factors for pneumonia in the elderly. Am J Med.

[4] Mannino DM, Davis KJ, Kiri VA (2009). Chronic obstructive pulmonary disease and hospitalizations for pneumonia in a US cohort. Respir Med.

[5] Polverino E, Torres Marti A (2011). Community-acquired pneumonia. Minerva Anestesiol.

[6] Cillóniz C, Polverino E, Ewig S, Aliberti S, Gabarrús A, Menéndez R, Mensa J, Blasi F, Torres A (2013). Impact of age and comorbidity on cause and outcome in community-acquired pneumonia. Chest.

[7] Torres A, Peetermans WE, Viegi G, Blasi F (2013). Risk factors for community-acquired pneumonia in adults in Europe: a literature review. Thorax.

[8] Blasi F, Mantero M, Santus P, Tarsia P (2012). Understanding the burden of pneumococcal disease in adults. Clin Microbiol Infect.

[9] Almirall J, Bolíbar I, Vidal J, Sauca G, Coll P, Niklasson B, Bartolomé M, Balanzó X (2000). Epidemiology of community-acquired pneumonia in adults: a population-based study. Eur Respir J.

[10] Ewig S, Birkner N, Strauss R, Schaefer E, Pauletzki J, Bischoff H, Schraeder P, Welte T, Hoeffken G (2009). New perspectives on community-acquired pneumonia in 388,406 patients. Results from a nationwide mandatory performance measurement programme in healthcare quality. Thorax.

[11] Trotter CL, Stuart JM, George R, Miller E (2008). Increasing hospital admissions for pneumonia, England. Emerg Infect Dis.

[12] Rozenbaum MH, Pechlivanoglou P, van der Werf TS, Lo-Ten-Foe JR, Postma MJ, Hak E (2013). The role of *Streptococcus pneumoniae* in community-acquired pneumonia among adults in Europe: a meta-analysis. Eur J Clin Microbiol Infect Dis.

[13] Weber D, Berger A, Edelsberg J, Huang X-Y, Oster G (2011) Distribution of pathogens in patients hospitalised for community-acquired pneumonia: analysis of data from US hospitals 2006–2009. In: Proceedings of the 49th Annual Meeting of the Infectious Diseases Society of America (IDSA), October 20–23, 2011, Boston, MA, USA

[14] Cunha BA (2008). Atypical pneumonias: current clinical concepts focusing on Legionnaires’ disease. Curr Opin Pulm Med.

[15] Defres S, Marwick C, Nathwani D (2009). MRSA as a cause of lung infection including airway infection, community-acquired pneumonia and hospital-acquired pneumonia. Eur Respir J.

[16] Strålin K, Söderquist B (2006). *Staphylococcus aureus* in community-acquired pneumonia. Chest.

[17] Vardakas KZ, Matthaiou DK, Falagas ME (2009). Incidence, characteristics and outcomes of patients with severe community acquired-MRSA pneumonia. Eur Respir J.

[18] Woodhead M, Blasi F, Ewig S, Garau J, Huchon G, Ieven M, Ortqvist A, Schaberg T, Torres A, van der Heijden G, Read R, Verheij TJ, Joint Taskforce of the European Respiratory Society and European Society for Clinical Microbiology and Infectious Diseases (2011). Guidelines for the management of adult lower respiratory tract infections—full version. Clin Microbiol Infect.

[19] Lim WS, Macfarlane JT, Boswell TC, Harrison TG, Rose D, Leinonen M, Saikku P (2001). Study of community acquired pneumonia aetiology (SCAPA) in adults admitted to hospital: implications for management guidelines. Thorax.

[20] Garcia-Vidal C, Fernández-Sabé N, Carratalà J, Díaz V, Verdaguer R, Dorca J, Manresa F, Gudiol F (2008). Early mortality in patients with community-acquired pneumonia: causes and risk factors. Eur Respir J.

[21] Lujan M, Gallego M, Fontanals D, Mariscal D, Rello J (2004). Prospective observational study of bacteremic pneumococcal pneumonia: effect of discordant therapy on mortality. Crit Care Med.

[22] Cillóniz C, Ewig S, Ferrer M, Polverino E, Gabarrús A, Puig de la Bellacasa J, Mensa J, Torres A (2011). Community-acquired polymicrobial pneumonia in the intensive care unit: aetiology and prognosis. Crit Care.

[23] Kothe H, Bauer T, Marre R, Suttorp N, Welte T, Dalhoff K, Competence Network for Community-Acquired Pneumonia study group (2008). Outcome of community-acquired pneumonia: influence of age, residence status and antimicrobial treatment. Eur Respir J.

[24] Almirall J, Rofes L, Serra-Prat M, Icart R, Palomera E, Arreola V, Clavé P (2013). Oropharyngeal dysphagia is a risk factor for community-acquired pneumonia in the elderly. Eur Respir J.

[25] Cabre M, Serra-Prat M, Palomera E, Almirall J, Pallares R, Clavé P (2010). Prevalence and prognostic implications of dysphagia in elderly patients with pneumonia. Age Ageing.

[26] Vila-Corcoles A, Ochoa-Gondar O, Rodriguez-Blanco T, Raga-Luria X, Gomez-Bertomeu F, EPIVAC Study Group (2009). Epidemiology of community-acquired pneumonia in older adults: a population-based study. Respir Med.

[27] Chidiac C, Che D, Pires-Cronenberger S, Jarraud S, Campèse C, Bissery A, Weinbreck P, Brun-Buisson C, Sollet JP, Ecochard R, Desenclos JC, Etienne J, Vanhems P; French Legionnaires’ Disease Study Group (2012) Factors associated with hospital mortality in community-acquired legionellosis in France. Eur Respir J 39:963–970 10.1183/09031936.00076911 22005914

[28] Sopena N, Force L, Pedro-Botet ML, Barrufet P, Sauca G, García-Núñez M, Tolchinsky G, Capdevila JA, Sabrià M (2007). Sporadic and epidemic community legionellosis: two faces of the same illness. Eur Respir J.

[29] Sopena N, Pedro-Botet L, Mateu L, Tolschinsky G, Rey-Joly C, Sabrià M (2007). Community-acquired legionella pneumonia in elderly patients: characteristics and outcome. J Am Geriatr Soc.

[30] Kofteridis D, Samonis G, Mantadakis E, Maraki S, Chrysofakis G, Alegakis D, Papadakis J, Gikas A, Bouros D (2009). Lower respiratory tract infections caused by Haemophilus influenzae: clinical features and predictors of outcome. Med Sci Monit.

[31] Ruiz LA, Gómez A, Jaca C, Martínez L, Gómez B, Zalacain R (2010). Bacteraemic community-acquired pneumonia due to Gram-negative bacteria: incidence, clinical presentation and factors associated with severity during hospital stay. Infection.

[32] von Baum H, Welte T, Marre R, Suttorp N, Ewig S, CAPNETZ study group (2010). Community-acquired pneumonia through Enterobacteriaceae and *Pseudomonas aeruginosa*: Diagnosis, incidence and predictors. Eur Respir J.

[33] Bénard A, Mercié P, Alioum A, Bonnet F, Lazaro E, Dupon M, Neau D, Dabis F, Chêne G, Groupe d’Epidémiologie Clinique du Sida en Aquitaine (2010). Bacterial pneumonia among HIV-infected patients: decreased risk after tobacco smoking cessation. ANRS CO3 Aquitaine Cohort, 2000–2007. PLoS One.

[34] Curran A, Falcó V, Crespo M, Martinez X, Ribera E, Villar del Saz S, Imaz A, Coma E, Ferrer A, Pahissa A (2008). Bacterial pneumonia in HIV-infected patients: use of the pneumonia severity index and impact of current management on incidence, aetiology and outcome. HIV Med.

[35] Le Moing V, Rabaud C, Journot V, Duval X, Cuzin L, Cassuto JP, Al Kaied F, Dellamonica P, Chêne G, Raffi F, APROCO Study Group (2006). Incidence and risk factors of bacterial pneumonia requiring hospitalization in HIV-infected patients started on a protease inhibitor-containing regimen. HIV Med.

[36] Madeddu G, Porqueddu EM, Cambosu F, Saba F, Fois AG, Pirina P, Mura MS (2008). Bacterial community acquired pneumonia in HIV-infected inpatients in the highly active antiretroviral therapy era. Infection.

[37] Manno D, Puoti M, Signorini L, Lapadula G, Cadeo B, Soavi L, Paraninfo G, Allegri R, Cristini G, Viale P, Carosi G (2009). Risk factors and clinical characteristics associated with hospitalization for community-acquired bacterial pneumonia in HIV-positive patients according to the presence of liver cirrhosis. Infection.

[38] Molinos L, Clemente MG, Miranda B, Alvarez C, del Busto B, Cocina BR, Alvarez F, Gorostidi J, Orejas C, ASTURPAR Group (2009). Community-acquired pneumonia in patients with and without chronic obstructive pulmonary disease. J Infect.

[39] Falguera M, Carratalà J, Ruiz-Gonzalez A, Garcia-Vidal C, Gazquez I, Dorca J, Gudiol F, Porcel JM (2009). Risk factors and outcome of community-acquired pneumonia due to Gram-negative bacilli. Respirology.

[40] Gutiérrez F, Masiá M, Rodríguez JC, Mirete C, Soldán B, Padilla S, Hernández I, De Ory F, Royo G, Hidalgo AM (2005). Epidemiology of community-acquired pneumonia in adult patients at the dawn of the 21st century: a prospective study on the Mediterranean coast of Spain. Clin Microbiol Infect.

[41] Klapdor B, Ewig S, Pletz MW, Rohde G, Schütte H, Schaberg T, Welte T, CAPNETZ Study Group (2012). Community-acquired pneumonia in younger patients is an entity on its own. Eur Respir J.

[42] Liapikou A, Polverino E, Ewig S, Cillóniz C, Marcos MA, Mensa J, Bello S, Martin-Loeches I, Menéndez R, Torres A (2012). Severity and outcomes of hospitalised community-acquired pneumonia in COPD patients. Eur Respir J.

[43] Carratalà J, Mykietiuk A, Fernández-Sabé N, Suárez C, Dorca J, Verdaguer R, Manresa F, Gudiol F (2007). Health care-associated pneumonia requiring hospital admission: epidemiology, antibiotic therapy, and clinical outcomes. Arch Intern Med.

[44] Cillóniz C, Ewig S, Polverino E, Muñoz-Almagro C, Marco F, Gabarrús A, Menéndez R, Mensa J, Torres A (2012). Pulmonary complications of pneumococcal community-acquired pneumonia: incidence, predictors, and outcomes. Clin Microbiol Infect.

[45] de Roux A, Cavalcanti M, Marcos MA, Garcia E, Ewig S, Mensa J, Torres A (2006). Impact of alcohol abuse in the etiology and severity of community-acquired pneumonia. Chest.

[46] Viasus D, Garcia-Vidal C, Castellote J, Adamuz J, Verdaguer R, Dorca J, Manresa F, Gudiol F, Carratalà J (2011). Community-acquired pneumonia in patients with liver cirrhosis: clinical features, outcomes, and usefulness of severity scores. Medicine (Baltimore).

[47] Viegi G, Pistelli R, Cazzola M, Falcone F, Cerveri I, Rossi A, Ugo Di Maria G (2006). Epidemiological survey on incidence and treatment of community acquired pneumonia in Italy. Respir Med.

[48] Garcia-Vidal C, Carratalà J, Fernández-Sabé N, Dorca J, Verdaguer R, Manresa F, Gudiol F (2009). Aetiology of, and risk factors for, recurrent community-acquired pneumonia. Clin Microbiol Infect.

[49] Giannella M, Pinilla B, Capdevila JA, Martínez Alarcón J, Muñoz P, López Álvarez J, Bouza E, Estudio de Neumonía En Medicina Interna study Group from the Sociedad Española de Medicina Interna (2012). Pneumonia treated in the internal medicine department: focus on healthcare-associated pneumonia. Clin Microbiol Infect.

[50] Viasus D, Garcia-Vidal C, Cruzado JM, Adamuz J, Verdaguer R, Manresa F, Dorca J, Gudiol F, Carratalà J (2011). Epidemiology, clinical features and outcomes of pneumonia in patients with chronic kidney disease. Nephrol Dial Transplant.

[51] Reissig A, Mempel C, Schumacher U, Copetti R, Gross F, Aliberti S (2013). Microbiological diagnosis and antibiotic therapy in patients with community-acquired pneumonia and acute COPD exacerbation in daily clinical practice: comparison to current guidelines. Lung.

[52] Blasi F, Garau J, Medina J, Avila M, McBride K, Ostermann H (2013). Current management of patients hospitalized with community-acquired pneumonia across Europe: outcomes from REACH. Respir Res.

[53] Mandell LA, Wunderink RG, Anzueto A, Bartlett JG, Campbell GD, Dean NC, Dowell SF, File TM, Musher DM, Niederman MS, Torres A, Whitney CG, Infectious Diseases Society of America, American Thoracic Society (2007). Infectious Diseases Society of America/American Thoracic Society consensus guidelines on the management of community-acquired pneumonia in adults. Clin Infect Dis.

[54] Jokinen C, Heiskanen L, Juvonen H, Kallinen S, Kleemola M, Koskela M, Leinonen M, Rönnberg PR, Saikku P, Stén M, Tarkiainen A, Tukiainen H, Pyörälä K, Mäkelä PH (2001). Microbial etiology of community-acquired pneumonia in the adult population of 4 municipalities in eastern Finland. Clin Infect Dis.

[55] Aliberti S, Cilloniz C, Chalmers JD, Zanaboni AM, Cosentini R, Tarsia P, Pesci A, Blasi F, Torres A (2013). Multidrug-resistant pathogens in hospitalised patients coming from the community with pneumonia: a European perspective. Thorax.

[56] Christensen D, Feldman C, Rossi P, Marrie T, Blasi F, Luna C, Fernandez P, Porras J, Martinez J, Weiss K, Levy G, Lode H, Gross P, File T, Ramirez J; Community-Acquired Pneumonia Organization Investigators (2005) HIV infection does not influence clinical outcomes in hospitalized patients with bacterial community-acquired pneumonia: results from the CAPO international cohort study. Clin Infect Dis 41:554–556 10.1086/432063 16028168

[57] Malinis M, Myers J, Bordon J, Peyrani P, Kapoor R, Nakamatzu R, Lopardo G, Torres A, Feldman C, Allen M, Arnold F, Ramirez J (2010). Clinical outcomes of HIV-infected patients hospitalized with bacterial community-acquired pneumonia. Int J Infect Dis.

[58] Lim WS, Baudouin SV, George RC, Hill AT, Jamieson C, Le Jeune I, Macfarlane JT, Read RC, Roberts HJ, Levy ML, Wani M, Woodhead MA, Pneumonia Guidelines Committee of the BTS Standards of Care Committee (2009). BTS guidelines for the management of community acquired pneumonia in adults: update 2009. Thorax.

[59] Menéndez R, Torres A, Aspa J, Capelastegui A, Prat C, Rodríguez de Castro F, Sociedad Española de Neumología y Cirugía Torácica (2010). Community acquired pneumonia. New guidelines of the Spanish Society of Chest Diseases and Thoracic Surgery (SEPAR). Arch Bronconeumol.

[60] Höffken G, Lorenz J, Kern W, Welte T, Bauer T, Dalhoff K, Dietrich E, Ewig S, Gastmeier P, Grabein B, Halle E, Kolditz M, Marre R, Sitter H, Paul-Ehrlich-Society of Chemotherapy, German Respiratory Diseases Society, German Infectious Diseases Society, Competence Network CAPNETZ for the Management of Lower Respiratory Tract Infections and Community-acquired Pneumonia (2010). Guidelines of the Paul-Ehrlich-Society of Chemotherapy, the German Respiratory Diseases Society, the German Infectious Diseases Society and of the Competence Network CAPNETZ for the Management of Lower Respiratory Tract Infections and Community-acquired Pneumonia. Pneumologie.

[61] Blasi F, Bulfoni A, Concia E, Costantino S, Di Rosa S, Iori I, Mazzei T, Schito GC (2002). Gestione delle infezioni delle basse vie respiratorie in medicina interna. GIMI.

[62] Blasi F, Bulfoni A, Concia E, Costantino S, Giusti M, Iori I, Mazzei T, Schito GC (2010). Attualità nella gestione delle infezioni delle basse vie respiratorie in medicina interna. Ital J Med.

[63] Torres A, Barberán J, Falguera M, Menéndez R, Molina J, Olaechea P, Rodríguez A, Grupo de la Guía Multidisciplinar para el Manejo de la Neumonía Adquirida en la Comunidad (2013). Multidisciplinary guidelines for the management of community-acquired pneumonia. Med Clin (Barc).

[64] European Centre for Disease Prevention and Control (ECDC) (2012) Antimicrobial resistance surveillance in Europe 2011. Annual Report of the European Antimicrobial Resistance Surveillance Network (EARS-Net). Available online at: http://www.ecdc.europa.eu/en/publications/Publications/antimicrobial-resistance-surveillance-europe-2011.pdf. Accessed 2 September 2013

[65] Holm A, Nexoe J, Bistrup LA, Pedersen SS, Obel N, Nielsen LP, Pedersen C (2007). Aetiology and prediction of pneumonia in lower respiratory tract infection in primary care. Br J Gen Pract.

[66] Migliorati PL, Boccoli E, Bracci LS, Sestini P, Melani AS (2006). A survey on hospitalised community-acquired pneumonia in Italy. Monaldi Arch Chest Dis.

[67] Pérez-Sola MJ, Torre-Cisneros J, Pérez-Zafrilla B, Carmona L, Descalzo MA, Gómez-Reino JJ, BIOBADASER Study Group (2011). Infections in patients treated with tumor necrosis factor antagonists: incidence, etiology and mortality in the BIOBADASER registry. Med Clin (Barc).

[68] Bewick T, Sheppard C, Greenwood S, Slack M, Trotter C, George R, Lim WS (2012). Serotype prevalence in adults hospitalised with pneumococcal non-invasive community-acquired pneumonia. Thorax.

