# Supplementary material for: The aetiology and antibiotic management of community-acquired pneumonia in adults in Europe: a literature review
Source: Eur J Clin Microbiol Infect Dis. 2014 Feb 15;33(7):1065–79. doi: 10.1007/s10096-014-2067-1 (PMC4042014; doi:10.1007/s10096-014-2067-1)
Supplement: Supplementary file 1 — (DOC 0.98 mb) [file 10096_2014_2067_MOESM1_ESM.doc]

**Supplementary table 1.** Microbiological techniques used for isolation of pathogens and frequency of pathogens in patients with CAP

| **Citation** | **Microbiological methodology** | **Population (*n*)** | **Pathogen** | **Study cohorts** | | | | |
| --- | --- | --- | --- | --- | --- | --- | --- | --- |
|  |  |  |  | **Frequency of pathogens**  ***n/N* (%)** | | | | |
| Almirall et al. 2013 [24] | - Blood cultures (*n*=30) - Urine antigen tests for *S. pneumoniae* (*n*=30) & *L. pneumophila* (*n*=33) - Respiratory samples: sputum (*n*=9), tracheal aspirate (*n*=1) and pleural fluid (*n*=2); sublingual smear (*n*=29) | Patients ≥70 yrs hospitalised with CAP (*n*=36) | Patients with pathogens identified | 20/36 (55.6) |  | | |  |
|  |  |  | *S. pneumoniae* | 17/20 (85) |  | | |  |
|  |  |  | *P. aeruginosa* | 1/20 (5) |  | | |  |
|  |  |  | *K. pneumoniae* | 1/20 (5) |  | | |  |
|  |  |  | *P. aeruginosa* & *K. pneumoniae* | 1/20 (5) |  | | |  |
| Bénard et al. 2010 [33] | Not reported | HIV patients with CAP (135 episodes) | Episodes with pathogens identified | 31/135 (23) |  | | |  |
|  |  |  | *S. pneumoniae* | 22/31 (71.0) |  | | |  |
|  |  |  | *Pseudomonas* | 6/31 (19.4) |  | | |  |
|  |  |  | *S. aureus* | 2/31 (6.5) |  | | |  |
|  |  |  | *H. influenzae* | 1/31 (3.2) |  | | |  |
| Bewick et al. 2012 [68] | - Urine antigen tests for *S. pneumoniae* - Blood, sputum and/or bronchoalveolar lavage cultures - Serotyping: multiplexed serotype-specific pneumococcal immunoassay and slide agglutination in bacterial pneumococcal patients | Patients ≥16 yrs hospitalised for CAP (*n*=920) | *S. pneumoniae* | 366/920 (39.8) |  | | |  |
|  |  |  | Serotype determined | 242/366 (66.1) |  |  |  |  |
|  |  |  | Serotype 14 | 45/242 (18.6) |  |  |  |  |
|  |  |  | Serotype 1 | 40/242 (16.5) |  |  |  |  |
|  |  |  | Serotype 8 | 35/242 (14.5) |  |  |  |  |
|  |  |  | Serotype 3 | 20/242 (8.3) |  |  |  |  |
|  |  |  | Serotype 19A | 20/242 (8.3) |  |  |  |  |
| Cabre et al. 2010 [25] | Not reported | Patients ≥70 yrs with CAP requiring hospitalisation (*n*=134) | Patients with pathogens identified | 17/134 (12.7) |  | | |  |
|  |  |  | Gram-negative (*P. aeruginosa, E. coli and S. marcescens*) | 6/17 (35.3) |  | | |  |
|  |  |  | *H. influenzae* | 5/17 (29.4) |  | | |  |
|  |  |  | *S. pneumoniae* | 4/17 (23.5) |  | | |  |
|  |  |  | *L. pneumophila* | 2/17 (11.8) |  | | |  |
| Carratalà et al. 2007 [43] | - Blood, normally sterile fluids and sputum samples - Urine antigen tests for *S. pneumoniae* & *L. pneumophila* - Serology | Patients with CAP requiring hospitalisation (*n*=601) | Patients with pathogens identified | 337/601 (56.1) |  | | |  |
|  |  |  | *S. pneumoniae* | 204/337 (60.5) |  | | |  |
|  |  |  | *L. pneumophila* | 53/337 (15.7) |  | | |  |
|  |  |  | *H. influenzae* | 36/337 (10.7) |  | | |  |
|  |  |  | *M. pneumoniae* | 12/337 (3.6) |  | | |  |
|  |  |  | *C. burnetii* | 7/337 (2.1) |  | | |  |
|  |  |  | *C. pneumoniae* | 5/337 (1.5) |  | | |  |
|  |  |  | *P. aeruginosa* | 3/337 (0.9) |  | | |  |
|  |  |  | *E. coli* | 2/337 (0.6) |  | | |  |
|  |  |  | *K. pneumoniae* | 1/337 (0.3) |  | | |  |
| Cillóniz et al. 2011 [22] | Samples:   - Sputum, urine, blood, nasopharyngeal swabs and, if available, pleural puncture, BAS and BAL fluid   Tests:   - Urine antigen tests for *S. pneumoniae* & *L. pneumophila* - Sputum, BAS and BAL specimens Gram-stained and Ziehl–Neelsen-stained for bacterial and mycobacteria detection, respectively - BAL: additional stains for fungal detection - Sputum and pleural fluid qualitatively cultured for bacterial pathogens, fungi and mycobacteria - BAS and BAL samples quantitatively cultured for bacterial pathogens, *Legionella* species, fungi and mycobacteria - Nasopharyngeal swab and BAL specimens: immunofluorescence assay and reverse transcriptase polymerase chain reaction for respiratory viruses - Serology: for *C. pneumoniae*, *C. burnetii* , *M. pneumoniae,* *L. pneumophila* and respiratory viruses (influenza viruses A and B, parainfluenza viruses 1 to 3, RSV and adenovirus) | Patients with CAP admitted to ICU (*n*=362) |  | **With lower respiratory tract (LRT) samples** | **No LRT samples (*n*=276)** | | |  |
|  |  |  |  | **(*n*=86)** |  |  |  |  |
|  |  |  | Aetiology defined | 60/86 (69.8) | 136/276 (49.3) | | | *p*=0.002 |
|  |  |  | *S. pneumoniae* | 29/60 (48.3) | 93/136 (68.4) | | |  |
|  |  |  | Virus | 10/60 (16.7) | 21/136 (15.4) | | |  |
|  |  |  | MRSA | 8/60 (13.3) | 6/136 (4.4) | | |  |
|  |  |  | *P. aeruginosa* | 7/60 (11.7) | 7/136 (5.1) | | |  |
|  |  |  | Gram-negative enteric bacilli | 5/60 (8.3) | 5/136 (3.7) | | |  |
|  |  |  | *H. influenzae* | 5/60 (8.3) | 3/136 (2.2) | | |  |
|  |  |  | *M. pneumoniae* | 4/60 (6.7) | 2/136 (1.5) | | |  |
|  |  |  | *S. aureus* | 3/60 (5) | 4/136 (2.9) | | |  |
|  |  |  | *L. pneumophila* | 1/60 (1.7) | 10/136 (7.4) | | |  |
|  |  |  | *C. pneumoniae* | 1/60 (1.7) | 5/136 (3.7) | | |  |
|  |  |  | *C. burnetii* | 1/60 (1.7) | 2/136 (1.5) | | |  |
|  |  |  | *S. viridans* | 1/60 (1.7) | 0 | | |  |
|  |  |  | 30-day mortality | 19/86 (22.1) | 18/276 (6.5) | | |  |
|  |  |  | Monomicrobial infection | 157/196 (80.1) | | | |  |
|  |  |  | Polymicrobial infection | 39/196 (19.9) | | | |  |
|  |  | [****p*<0.001 ***p*<0.01 **p*<0.05 monomicrobial versus polymicrobial] |  | **Monomicrobial aetiology (*n*=157)** | | **Polymicrobial aetiology (*n*=39)** | | **Two pathogens (*n*=33)** |
|  |  |  | *S. pneumoniae* | 94/157 (60) | | 28/39 (72) | | 23/33 (70) |
|  |  |  | Respiratory virus | 16/157 (10) | | 15/39 (39)*** | | 14/33 (42) |
|  |  |  | *L. pneumophila* | 10/157 (6) | | 1/39 (3) | | 1/33 (3) |
|  |  |  | *P. aeruginosa* | 6/157 (4) | | 8/39 (21)*** | | 6/33 (18) |
|  |  |  | Gram-negative enteric bacilli | 6/157 (4) | | 7/39 (18)** | | 5/33 (15) |
|  |  |  | *S. aureus (MRSA)* | 5/157 (3) | | 9/39 (23)*** | | 5/33 (15) |
|  |  |  | *S. aureus (MSSA)* | 4/157 (3) | | 3/39 (8) | | 2/33 (6) |
|  |  |  | *H. influenzae* | 4/157 (3) | | 4/39 (10)* | | 3/33 (9) |
|  |  |  | *M. pneumoniae* | 4/157 (3) | | 2/39 (5) | | 2/33 (6) |
|  |  |  | *C. pneumoniae* | 4/157 (3) | | 2/39 (5) | | 1/33 (3) |
|  |  |  | *C. burnetii* | 2/157 (1) | | 1/39 (3) | | 1/33 (3) |
|  |  |  | *M. catarrhalis* | 1/157 (1) | | 2/39 (5)* | | 1/33 (3) |
|  |  |  | *S. pyogenes* | 1/157 (1) | | 1/39 (3) | | 1/33 (3) |
|  |  |  | *S. viridans* | 0 | | 1/39 (3) | | 1/33 (3) |
| Curran et al. 2008 [34] | - Blood cultures (all patients), sputum sample when available, pleural fluid - Urine antigen for *L. pneumophila* if clinical or epidemiological suspicion of legionellosis; and in all cases of severe CAP | HIV patients hospitalised for CAP (*n*=161; 186 episodes) | Episodes with pathogens identified | 102/186 (54.8) | |  | |  |
|  |  |  | *S. pneumoniae* | 59/102 (57.8) | |  | |  |
|  |  |  | *L. pneumophila* | 11/102 (10.8) | |  | |  |
|  |  |  | Non-pseudomonal Gram-negative enteric bacilli | 8/102 (7.8) | |  | |  |
|  |  |  | *H. influenzae* | 6/102 (5.9) | |  | |  |
|  |  |  | *P. aeruginosa* | 6/102 (5.9) | |  | |  |
|  |  |  | Others | 12/102 (11.8) | |  | |  |
|  |  | Stratified by CD4 cell count |  | **<200 cells/µl**  **(*n*=84)** | | **200–350 cells/µl**  **(*n*=55)** | | **>350 cells/µl (*n*=41)** |
|  |  |  | *S. pneumoniae* | 25/84 (29.8) | | 18/55 (32.7) | | 14/41 (34.1) |
|  |  |  | *P. aeruginosa* | 3/84 (3.6) | | 0 | | 2/41 (4.9) |
|  |  |  | *L. pneumophila* | 2/84 (2.4) | | 3/55 (5.5) | | 5/41 (12.2) |
|  |  | Stratified by severity |  | **Low-risk PSI I-III**  **(*n*=123)** | | **High-risk PSI IV-V**  **(*n*=54)** | |  |
|  |  |  | *S. pneumoniae* | 36/123 (29.3) | | 23/54 (42.6) | |  |
|  |  |  | *L. pneumophila* | 7/123 (5.7) | | 4/54 (7.4) | |  |
|  |  |  | *P. aeruginosa* | 4/123 (3.3) | | 2/54 (3.7) | |  |
| de Roux et al. 2006 [45] | - Cultures from sputum, blood, pleural fluid (when available) and transthoracic needle aspiration sample - Urinary antigen tests for *L. pneumophila* and *S. pneumoniae* - Tracheobronchial aspiration, BAL fluid and protected-specimen brush - Paired serology (at hospital admission and within the third to sixth weeks thereafter) for the following: *C. pneumoniae*, *L. pneumophila*, *C. burnetii,* *M. pneumoniae*, respiratory virus (i.e., influenza virus A and B), parainfluenza virus 1 to 3, respiratory syncytial virus and adenovirus | Patients hospitalised for CAP (current alcohol abuse = daily consumption >80 g for men and 60 g for women during the past 2 yrs; former alcohol abuse = no abuse in past 1 yr) |  | **No abuse**  **(*n*=1,165)** | |  | | **Former abuse**  **(*n*=54)** |
|  |  |  |  |  |  |  |  |  |
|  |  |  | Patients with pathogens identified | 442/1,165 (38) | | 63/128 (49) | | 28/54 (52) |
|  |  |  | *S. pneumoniae* | 187/442 (42.3) | | 34/63 (54.0) | | 16/28 (57.1) |
|  |  |  | Gram-negative enteric bacilli | 123/442 (27.8) | | 12/63 (19.0) | | 7/28 (25) |
|  |  |  | *H. influenzae* | 50/442 (11.3) | | 2/63 (3.2) | | 3/28 (10.7) |
|  |  |  | Virus | 47/442 (10.6) | | 9/63 (14.3) | | 0 |
|  |  |  | *P. aeruginosa* | 39/442 (8.8) | | 6/63 (9.5) | | 3/28 (10.7) |
|  |  |  | *C. pneumoniae* | 37/442 (8.4) | | 6/63 (9.5) | | 2/28 (7.1) |
|  |  |  | *L. pneumophila* | 37/442 (8.4) | | 5/63 (7.9) | | 2/28 (7.1) |
|  |  |  | *M. pneumoniae* | 25/442 (5.7) | | 2/63 (3.2) | | 1/28 (3.6) |
|  |  |  | *C. burnetii* | 5/442 (1.1) | | 2/63 (3.2) | | 0 |
| Falguera 2009 [39] | - Two blood cultures - Gram stain and sputum culture - Gram stain and pleural fluid culture - Paired serum samples, obtained at presentation and 4–8 weeks later, for serological studies to detect antibodies against *M. pneumoniae*, *C. pneumoniae*, *C. psittaci* and *C. burnetii* - Urinary antigen test for *S. pneumoniae* and *L. pneumophila* - In some cases, invasive techniques, mainly transthoracic needle aspiration | Patients hospitalised with CAP (*n*=3,272) | Patients with pathogens identified | 1,857/3,272 (57) | |  | |  |
|  |  |  | *S. pneumoniae* | 972/1,857 (52.3) | |  | |  |
|  |  |  | *L. pneumophila* | 212/1,857 (11.4) | |  | |  |
|  |  |  | *H. influenzae* | 137/1,857 (7.4) | |  | |  |
|  |  |  | *C. pneumoniae* | 95/1,857 (5.1) | |  | |  |
|  |  |  | *M. pneumoniae* | 89/1,857 (4.8) | |  | |  |
|  |  |  | 30-day mortality | 236/3,211 (7) | |  | |  |
|  |  |  | Definite and probable diagnoses |  | |  | |  |
|  |  | Patients hospitalised with CAP – Gram-negative infections (*n*=61; 63 infections) | *P. aeruginosa* | 29/1,857 (1.6) | |  | |  |
|  |  |  | *E. coli* | 17/1,857 (0.9) | |  | |  |
|  |  |  | *K. pneumoniae* | 7/1,857 (0.4) | |  | |  |
|  |  |  | *Enterobacter* spp. | 3/1,857 (0.2) | |  | |  |
|  |  |  | *S. marcescens* | 2/1,857 (0.1) | |  | |  |
|  |  |  | *C. freundii* | 1/1,857 (0.1) | |  | |  |
|  |  |  | *K. oxytoca* | 1/1,857 (0.1) | |  | |  |
|  |  |  | *Acinetobacter* sp. | 1/1,857 (0.1) | |  | |  |
|  |  |  | *A. hydrophila* | 1/1,857 (0.1) | |  | |  |
|  |  |  | *P. mirabilis* | 1/1,857 (0.1) | |  | |  |
|  |  |  | Mixed infection | 7/1,857 (0.4) | |  | |  |
|  |  |  | 30-day mortality | 22/1,857 (1.2) | |  | |  |
| Garcia-Vidal et al. 2009 [48] | Samples:   - Blood, normally sterile fluids, sputum and other samples   Tests:   - Urinary antigen tests for *S. pneumoniae* and *L. pneumophila* - Serology: to determine antibodies against the following pathogens: *M. pneumoniae* (indirect agglutination), *C. psittaci* (immunofluorescence [IF]), *C. pneumoniae* (micro-IF), *C. burnetii* (IF), *L. pneumophila* (serogroups 1–6) (enzyme immunoassay [EIA]), RSV (EIA), parainfluenza 3 virus (EIA) and influenza A virus (EIA) | Patients hospitalised with CAP |  | **Recurrent CAP**  **(*n*=146; 224 episodes)** | | **Non-recurrent CAP (*n*=1,410 patients/episodes)** | |  |
|  |  | [**p*<0.05 versus non-recurrent CAP] |  |  |  |  |  |  |
|  |  |  | Episodes with pathogens identified | 125/224 (55.8) | | 689/1,410 (48.9) | |  |
|  |  |  | *S. pneumoniae* | 63/125 (50.4) | | 366/689 (53.1) | |  |
|  |  |  | *H. influenzae* | 24/125 (19.2)* | | 85/689 (12.3) | |  |
|  |  |  | Atypical agents (*C. pneumoniae, C. psittaci, M. pneumoniae, C. burnetii)* | 7/125 (5.6) | | 75/689 (10.9) | |  |
|  |  |  | Gram-negative bacilli | 6/125 (4.8)* | | 12/689 (1.7) | |  |
|  |  |  | *L. pneumophila* | 4/125 (3.2)* | | 104/689 (15.1) | |  |
|  |  |  | Virus | 0 | | 5/689 (0.7) | |  |
| Giannella et al. 2012 [49] | - Blood culture, sputum, bronchial aspirate, bronchoalveolar lavage and pulmonary biopsy - Urinary antigen tests for *S. pneumoniae* and *L. pneumophila* - Serology - Nasopharyngeal swab for detection of the influenza virus | Patients with CAP treated in internal medicine departments (*n*=591) | Patients with pathogens identified | 148/591 (25) | |  | |  |
|  |  |  | *S. pneumoniae* | 94/148 (63.5) | |  | |  |
|  |  |  | *Enterobacteriaceae* | 17/148 (11.5) | |  | |  |
|  |  |  | *L. pneumophila* | 11/148 (7.4) | |  | |  |
|  |  |  | *H. influenzae* | 5/148 (3.4) | |  | |  |
|  |  |  | *P. aeruginosa* | 5/148 (3.4) | |  | |  |
|  |  |  | *S. aureus* | 2/148 (1.4) | |  | |  |
|  |  |  | Virus | 2/148 (1.4) | |  | |  |
| Gutiérrez et al. 2005 [40] | - Sputum samples for Gram’s stain and culture (patients with productive cough) - Two blood samples for culture (patients with fever ≥38°C) - Urinary antigen tests for *S. pneumoniae* and *L. pneumophila* (all patients, before starting antibiotic therapy) - Pleural fluid sample - Serum samples for serological testing, drawn during the acute and convalescent phases of illness - Bronchoscopic samples obtained were also cultured - Complement fixation (CF) test to detect antibodies against *M. pneumoniae*, *Chlamydia* spp., *C. burnetii*, influenza viruses A and B, respiratory syncytial virus and adenovirus - Indirect immunofluorescence test to detect antibodies against *L. pneumophila* - Microimmunofluorescence test to detect antibodies against *C. pneumoniae*, *C. psittaci* and *C. trachomatis* | Patients with CAP diagnosed in a single hospital; total population (*n*=493) | Patients with pathogens identified | 250/493 (50.7) | |  | |  |
|  |  |  | *S. pneumoniae* | 83/250 (33.2) | |  | |  |
|  |  |  | *M. pneumoniae* | 38/250 (15.2) | |  | |  |
|  |  |  | *Chlamydia* spp. | 30/250 (12) | |  | |  |
|  |  |  | *L. pneumophila* | 21/250 (8.4) | |  | |  |
|  |  |  | Virus | 20/250 (8) | |  | |  |
|  |  |  | Gram-negative, including *Pseudomonas* spp. | 16/250 (6.4) | |  | |  |
|  |  |  | *H. influenzae* | 9/250 (3.6) | |  | |  |
|  |  |  | *S. aureus* | 2/250 (0.8) | |  | |  |
|  |  |  | *C. burnetii* | 2/250 (0.8) | |  | |  |
|  |  |  | *M. catarrhalis* | 1/250 (0.4) | |  | |  |
|  |  |  | Mixed aetiology | 28/250 (11.2) | |  | |  |
|  |  | Patients with CAP diagnosed in a single hospital; patients with COPD (*n*=99) | Patients with pathogens identified | 48/99 (48.5) | |  | |  |
|  |  |  | *S. pneumoniae* | 18/48 (37.5) | |  | |  |
|  |  |  | Gram-negative, including *Pseudomonas* spp. | 8/48 (16.7) | |  | |  |
|  |  |  | *L. pneumophila* | 6/48 (12.5) | |  | |  |
|  |  |  | *Chlamydia* spp. | 2/48 (4.2) | |  | |  |
|  |  |  | *H. influenzae* | 2/48 (4.2) | |  | |  |
|  |  |  | *M. pneumoniae* | 2/48 (4.2) | |  | |  |
|  |  |  | Virus | 2/48 (4.2) | |  | |  |
|  |  |  | *M. catarrhalis* | 1/48 (2.1) | |  | |  |
|  |  |  | *C. burnetii* | 0 | |  | |  |
|  |  |  | *S. aureus* | 0 | |  | |  |
|  |  |  | Mixed aetiology | 7/48 (14.6) | |  | |  |
|  |  | Patients with CAP diagnosed in a single hospital; patients without comorbidity (*n*=264)  [**p*=0.01,  ***p*=0.001 compared with patients with underlying conditions] | Patients with pathogens identified | 130/264 (49.2) | |  | |  |
|  |  |  | *S. pneumoniae* | 35/130 (26.9) | |  | |  |
|  |  |  | *M. pneumoniae* | 30/130 (23.1)** | |  | |  |
|  |  |  | *Chlamydia* spp. | 23/130 (17.7)* | |  | |  |
|  |  |  | *L. pneumophila* | 11/130 (8.5) | |  | |  |
|  |  |  | Virus | 10/130 (7.7) | |  | |  |
|  |  |  | *H. influenzae* | 5/130 (3.8) | |  | |  |
|  |  |  | Gram-negative, including *Pseudomonas* spp. | 4/130 (3.1) | |  | | |
|  |  |  | *C. burnetii* | 2/130 (1.5) | |  |  |  |
|  |  |  | *S. aureus* | 1/130 (0.8) | |  |  |  |
|  |  |  | *M. catarrhalis* | 0 | |  |  |  |
|  |  |  | Mixed aetiology | 9/130 (6.9) | |  |  |  |
|  |  | Patients with CAP diagnosed in a single hospital; patients with underlying conditions (*n*=227) | Patients with pathogens identified | 118/227 (52.0) | |  |  | |
|  |  |  | *S. pneumoniae* | 48/118 (40.7) | |  |  | |
|  |  |  | Gram-negative, including *Pseudomonas* spp. | 12/118 (10.2) | |  |  | |
|  |  |  | *L. pneumophila* | 10/118 (8.5) | |  |  | |
|  |  |  | Virus | 10/118 (8.5) | |  |  | |
|  |  |  | *M. pneumoniae* | 8/118 (6.8) | |  |  | |
|  |  |  | *Chlamydia* spp. | 7/118 (5.9) | |  |  | |
|  |  |  | *H. influenzae* | 4/118 (3.4) | |  |  | |
|  |  |  | *M. catarrhalis* | 1/118 (0.8) | |  |  | |
|  |  |  | *C. burnetii* | 0 | |  |  | |
|  |  |  | *S. aureus* | 0 | |  |  | |
|  |  |  | Mixed aetiology | 18/118 (15.3) | |  |  | |
|  |  | Patients with CAP diagnosed in a single hospital; patients with diabetes mellitus (*n*=98) | Patients with pathogens identified | 51/98 (52.0) | |  |  | |
|  |  |  | *S. pneumoniae* | 22/51 (43.1) | |  |  | |
|  |  |  | *Chlamydia* spp. | 6/51 (11.8) | |  |  | |
|  |  |  | *M. pneumoniae* | 6/51 (11.8) | |  |  | |
|  |  |  | Virus | 6/51 (11.8) | |  |  | |
|  |  |  | *L. pneumophila* | 5/51 (9.8) | |  |  | |
|  |  |  | Gram-negative, including *Pseudomonas* spp. | 3/51 (5.9) | |  |  | |
|  |  |  | *H. influenzae* | 1/51 (2.0) | |  |  | |
|  |  |  | *C. burnetii* | 0 | |  |  | |
|  |  |  | *M. catarrhalis* | 0 | |  |  | |
|  |  |  | *S. aureus* | 0 | |  |  | |
|  |  |  | Mixed aetiology | 1/51(2.0) | |  |  | |
|  |  | Patients with CAP diagnosed in a single hospital; patients with dementia (*n*=52) | Patients with pathogens identified | 29/52 (55.8) | |  |  | |
|  |  |  | *S. pneumoniae* | 11/29 (37.9) | |  |  | |
|  |  |  | Virus | 4/29 (13.8) | |  |  | |
|  |  |  | Gram-negative, including *Pseudomonas* spp. | 3/29 (10.3) | |  |  | |
|  |  |  | *M. pneumoniae* | 2/29 (6.9) | |  |  | |
|  |  |  | *Chlamydia* spp. | 1/29 (3.4) | |  |  | |
|  |  |  | *L. pneumophila* | 1/29 (3.4) | |  |  | |
|  |  |  | *C. burnetii* | 0 | |  |  | |
|  |  |  | *H. influenzae* | 0 | |  |  | |
|  |  |  | *M. catarrhalis* | 0 | |  |  | |
|  |  |  | *S. aureus* | 0 | |  |  | |
|  |  |  | Mixed aetiology | 7/29 (24.1) | |  |  | |
|  |  | Patients with CAP diagnosed in a single hospital; patients aged 15–44 yrs (*n*=161)  [***p*<0.001 versus rest of patients] | Patients with pathogens identified | 88/161 (54.7) | |  |  | |
|  |  |  | *M. pneumoniae* | 28/88 (31.8)** | |  |  | |
|  |  |  | *S. pneumoniae* | 23/88 (26.1) | |  |  | |
|  |  |  | *Chlamydia* spp. | 10/88 (11.4) | |  |  | |
|  |  |  | Virus | 5/88 (5.7) | |  |  | |
|  |  |  | *L. pneumophila* | 4/88 (4.5) | |  |  | |
|  |  |  | *C. burnetii* | 2/88 (2.3) | |  |  | |
|  |  |  | Gram-negative, including *Pseudomonas* spp. | 2/88 (2.3) | |  |  | |
|  |  |  | *H. influenzae* | 2/88 (2.3) | |  |  | |
|  |  |  | *S. aureus* | 2/88 (2.3) | |  |  | |
|  |  |  | *M. catarrhalis* | 0 | |  |  | |
|  |  |  | Mixed aetiology | 10/88 (11.4) | |  |  | |
|  |  | Patients with CAP diagnosed in a single hospital; patients aged 45–64 yrs (*n*=109) | Patients with pathogens identified | 59/109 (54.1) | |  |  | |
|  |  |  | *S. pneumoniae* | 17/59 (28.8) | |  |  | |
|  |  |  | *Chlamydia* spp. | 12/59 (20.3) | |  |  | |
|  |  |  | *L. pneumophila* | 10/59 (16.9) | |  |  | |
|  |  |  | Virus | 5/59 (8.5) | |  |  | |
|  |  |  | *H. influenzae* | 4/59 (6.8) | |  |  | |
|  |  |  | *M. pneumoniae* | 3/59 (5.1) | |  |  | |
|  |  |  | Gram-negative, including *Pseudomonas* spp. | 2/59 (3.4) | |  |  | |
|  |  |  | *C. burnetii* | 0 | |  |  | |
|  |  |  | *M. catarrhalis* | 0 | |  |  | |
|  |  |  | *S. aureus* | 0 | |  |  | |
|  |  |  | Mixed aetiology | 6/59 (10.2%) | |  |  | |
|  |  | Patients with CAP diagnosed in a single hospital; patients aged 65–74 yrs (*n*=87) | Patients with pathogens identified | 43/87 (49.4) | |  |  | |
|  |  |  | *S. pneumoniae* | 17/43 (39.5) | |  |  | |
|  |  |  | *L. pneumophila* | 5/43 (11.6) | |  |  | |
|  |  |  | Virus | 5/43 (11.6) | |  |  | |
|  |  |  | *M. pneumoniae* | 4/43 (9.3) | |  |  | |
|  |  |  | *Chlamydia* spp. | 3/43 (7.0) | |  |  | |
|  |  |  | Gram-negative, including *Pseudomonas* spp. | 3/43 (7.0) | |  |  | |
|  |  |  | *H. influenzae* | 2/43 (4.7) | |  |  | |
|  |  |  | *M. catarrhalis* | 1/43 (2.3) | |  |  | |
|  |  |  | *C. burnetii* | 0 | |  |  | |
|  |  |  | *S. aureus* | 0 | |  |  | |
|  |  |  | Mixed aetiology | 3/43 (7.0) | |  |  | |
|  |  | Patients with CAP diagnosed in a single hospital; patients aged ≥75 yrs (*n*=136)  [**p*=0.007 versus rest of patients] | Patients with pathogens identified | 60/136 (44.1) | |  |  | |
|  |  |  | *S. pneumoniae* | 26/60 (43.3) | |  |  | |
|  |  |  | Gram-negative, including *Pseudomonas* spp. | 9/60 (15) | |  |  | |
|  |  |  | *Chlamydia* spp. | 5/60 (8.3) | |  |  | |
|  |  |  | Virus | 5/60 (8.3) | |  |  | |
|  |  |  | *M. pneumoniae* | 3/60 (5) | |  |  | |
|  |  |  | *L. pneumophila* | 2/60 (3.3)* | |  |  | |
|  |  |  | *H. influenzae* | 1/60 (1.7) | |  |  | |
|  |  |  | *C. burnetii* | 0 | |  |  | |
|  |  |  | *M. catarrhalis* | 0 | |  |  | |
|  |  |  | *S. aureus* | 0 | |  |  | |
|  |  |  | Mixed aetiology | 9/60 (15.0) | |  |  | |
|  |  | Patients with CAP diagnosed in a single hospital; patients classed as low severity (PSI I-III) (*n*=370) | Patients with pathogens identified | 183/370 (49.5) | |  |  | |
|  |  |  | *S. pneumoniae* | 55/183 (30.1) | |  |  | |
|  |  |  | *M. pneumoniae* | 33/183 (18.0) | |  |  | |
|  |  |  | *Chlamydia* spp. | 27/183 (14.8) | |  |  | |
|  |  |  | *L. pneumophila* | 15/183 (8.2) | |  |  | |
|  |  |  | Virus | 15/183 (8.2) | |  |  | |
|  |  |  | Gram-negative, including *Pseudomonas* spp. | 9/183 (4.9) | |  |  | |
|  |  |  | *H. influenzae* | 7/183 (3.8) | |  |  | |
|  |  |  | *S. aureus* | 2/183 (1.1) | |  |  | |
|  |  |  | *C. burnetii* | 2/183 (1.1) | |  |  | |
|  |  |  | *M. catarrhalis* | 1/183 (0.5) | |  |  | |
|  |  |  | Mixed aetiology | 17/183 (9.3) | |  |  | |
|  |  | Patients with CAP diagnosed in a single hospital; patients classed as high severity (PSI IV/V) (*n*=123) | Patients with pathogens identified | 67/123 (54.5) | |  |  | |
|  |  |  | *S. pneumoniae* | 28/67 (41.8) | |  |  | |
|  |  |  | Gram-negative, including *Pseudomonas* spp. | 7/67 (10.4) | |  |  | |
|  |  |  | *L. pneumophila* | 6/67 (9.0) | |  |  | |
|  |  |  | *M. pneumoniae* | 5/67 (7.5) | |  |  | |
|  |  |  | Virus | 5/67 (7.5) | |  |  | |
|  |  |  | *Chlamydia* spp. | 3/67 (4.5) | |  |  | |
|  |  |  | *H. influenzae* | 2/67 (3.0) | |  |  | |
|  |  |  | *C. burnetii* | 0 | |  |  | |
|  |  |  | *M. catarrhalis* | 0 | |  |  | |
|  |  |  | *S. aureus* | 0 | |  |  | |
|  |  |  | Mixed aetiology | 11/67 (16.4) | |  |  | |
|  |  | Patients with CAP diagnosed in a single hospital; patients hospitalised (*n*=361) | Patients with pathogens identified | 186/361 (51.5) | |  |  | |
|  |  |  | *S. pneumoniae* | 64/186 (34.4) | |  |  | |
|  |  |  | *M. pneumoniae* | 21/186 (11.3) | |  |  | |
|  |  |  | *Chlamydia* spp. | 17/186 (9.1) | |  |  | |
|  |  |  | *L. pneumophila* | 17/186 (9.1) | |  |  | |
|  |  |  | Gram-negative, including *Pseudomonas* spp. | 15/186 (8.1) | |  |  | |
|  |  |  | Virus | 14/186 (7.5) | |  |  | |
|  |  |  | *H. influenzae* | 6/186 (3.2) | |  |  | |
|  |  |  | *S. aureus* | 2/186 (1.1) | |  |  | |
|  |  |  | *C. burnetii* | 2/186 (1.1) | |  |  | |
|  |  |  | *M. catarrhalis* | 1/186 (0.5) | |  |  | |
|  |  |  | Mixed aetiology | 23/186 (12.4) | |  |  | |
|  |  | Patients with CAP diagnosed in a single hospital; outpatients (*n*=132)  [**p*=0.009 versus hospitalised patients] | Patients with pathogens identified | 70/132 (53.0) | |  |  | |
|  |  |  | *S. pneumoniae* | 19/70 (27.1) | |  |  | |
|  |  |  | *M. pneumoniae* | 17/70 (24.3)* | |  |  | |
|  |  |  | *Chlamydia* spp. | 13/70 (18.6) | |  |  | |
|  |  |  | Virus | 6/70 (8.6) | |  |  | |
|  |  |  | *L. pneumophila* | 4/70 (5.7) | |  |  | |
|  |  |  | *H. influenzae* | 3/70 (4.3) | |  |  | |
|  |  |  | Gram-negative, including *Pseudomonas* spp. | 1/70 (1.4) | |  |  | |
|  |  |  | *C. burnetii* | 0 | |  |  | |
|  |  |  | *M. catarrhalis* | 0 | |  |  | |
|  |  |  | *S. aureus* | 0 | |  |  | |
|  |  |  | Mixed aetiology | 5/70 (7.1) | |  |  | |
| Holm et al. 2007 [65] | - Blood and sputum cultured - Sputum analysed with polymerase chain reaction for influenza A and B viruses, RSV, parainfluenza virus type 3, adenovirus, rhinovirus, human metapneumovirus, *L. pneumophila*, *M. pneumoniae* and *C. pneumoniae/psittaci* | Patients with LRTI diagnosed in general practice |  | **Confirmed pneumonia (*n*=48)** | |  |  | |
|  |  |  | Patients with pathogens identified | 21/48 (44) | |  |  | |
|  |  |  | *S. pneumoniae* | 7/21 (33.3) | |  |  | |
|  |  |  | Virus | 6/21 (28.6) | |  |  | |
|  |  |  | *M. pneumoniae* | 4/21 (19.0) | |  |  | |
|  |  |  | *H. influenzae* | 2/21 (9.5) | |  |  | |
|  |  |  | *S. aureus* | 1/21 (4.8) | |  |  | |
| Klapdor et al. 2012 [41] | - Sputum and/or other respiratory secretions - Blood cultures - Urinary antigen tests for *S. pneumoniae* and *L. pneumophila* - Serology - Nasal and pharyngeal swabs - Investigations for viruses were performed only until July 2007 | Patients with CAP  [***p*<0.01, ****p*<0.001 versus patients ≥65 yrs] |  | **Age <65 yrs**  **(*n*=4,083)** | | **Age ≥65 yrs**  **(*n*=3,720)** |  | |
|  |  |  | Patients with pathogens identified | 1,338/4,083 (32.8)*** | | 993/3,720 (26.7) |  | |
|  |  |  | *S. pneumoniae* | 375/1,338 (28.0)** | | 330/993 (33.2) |  | |
|  |  |  | *M. pneumoniae* | 345/1,338 (25.1)*** | | 45/993 (4.5) |  | |
|  |  |  | *Legionella* spp. | 177/1,338 (13.2) | | 142/993 (14.3) |  | |
|  |  |  | *H. influenzae* | 86/1,338 (6.4) | | 53/993 (5.3) |  | |
|  |  |  | Virus | 64/1,338 (4.8)** | | 78/993 (7.8) |  | |
|  |  |  | Enterobacteria | 52/1,338 (3.9)*** | | 98/993 (9.9) |  | |
|  |  |  | *S. aureus* | 51/1,338 (3.8) | | 38/993 (3.8) |  | |
|  |  |  | *P. aeruginosa* | 16/1,338 (1.2)** | | 28/993 (2.8) |  | |
|  |  |  | *M. catarrhalis* | 9/1,338 (0.7) | | 12/993 (1.2) |  | |
|  |  |  | *C. pneumoniae* | 5/1,338 (0.4) | | 0 |  | |
|  |  | [***p*<0.01, ****p*<0.001 versus patients ≥50 yrs] |  | **Age <50 yrs**  **(*n*=2,293)** | | **Age ≥50 yrs**  **(*n*=5,510)** |  | |
|  |  |  | Patients with pathogens identified | 805/2,293 (35.1) | | 1,526/5,510 (27.7) |  | |
|  |  |  | *M. pneumoniae* | 299/805 (37.1)*** | | 91/1,526 (6.0) |  | |
|  |  |  | *S. pneumoniae* | 209/805 (26.0) | | 496/1,526 (32.5) |  | |
|  |  |  | *Legionella* spp. | 89/805 (11.1)** | | 230/1,526 (15.1) |  | |
|  |  |  | *H. influenzae* | 51/805 (6.3) | | 88/1,526 (5.8) |  | |
|  |  |  | Virus | 33/805 (4.1) | | 106/1,526 (6.9) |  | |
|  |  |  | *S. aureus* | 26/805 (3.2) | | 63/1,526 (4.1) |  | |
|  |  |  | Enterobacteria | 12/805 (1.5)*** | | 138/1,526 (9.0) |  | |
|  |  |  | *Pseudomonas* spp. | 6/805 (0.7)** | | 38/1,526 (2.5) |  | |
|  |  |  | *C. pneumoniae* | 4/805 (0.5) | | 1/1,526 (0.1) |  | |
|  |  |  | *M. catarrhalis* | 3/805 (0.4) | | 18/1,526 (1.2) |  | |
|  |  | [***p*<0.01, ****p*<0.001 versus patients ≥40 yrs] |  | **Age <40 yrs**  **(*n*=1,338)** | | **Age ≥40 yrs**  **(*n*=6,465)** |  | |
|  |  |  | Patients with pathogens identified | 477/1,338 (35.7) | | 1,854/6,465 (28.7) |  | |
|  |  |  | *M. pneumoniae* | 211/477 (44.2)*** | | 179/1,854 (9.7) |  | |
|  |  |  | *S. pneumoniae* | 118/477 (24.7)** | | 587/1,854 (31.7) |  | |
|  |  |  | *Legionella* spp. | 43/477 (9.0)** | | 276/1,854 (14.9) |  | |
|  |  |  | *H. influenzae* | 33/477 (6.9) | | 106/1,854 (5.7) |  | |
|  |  |  | *S. aureus* | 17/477 (3.6) | | 72/1,854 (3.9) |  | |
|  |  |  | Virus | 17/477 (3.6) | | 122/1,854 (6.6) |  | |
|  |  |  | Enterobacteria | 8/477 (1.7)*** | | 142/1,854 (7.7) |  | |
|  |  |  | *C. pneumoniae* | 4/477 (0.8)** | | 1/1,854 (0.1) |  | |
|  |  |  | *Pseudomonas* spp. | 3/477 (0.6) | | 41/1,854 (2.2) |  | |
|  |  |  | *M. catarrhalis* | 2/477 (0.4) | | 19/1,854 (1.0) |  | |
|  |  | [***p*<0.01, ****p*<0.001 versus patients ≥30 yrs] |  | **Age <30 yrs**  **(*n*=503)** | | **Age ≥30 yrs**  **(*n*=7,300)** |  | |
|  |  |  | Patients with pathogens identified | 179/503 (35.6) | | 2,152/7,300 (29.5) |  | |
|  |  |  | *M. pneumoniae* | 97/179 (54.2)*** | | 293/2,152 (13.6) |  | |
|  |  |  | *S. pneumoniae* | 35/179 (19.6)** | | 670/2,152 (31.1) |  | |
|  |  |  | *Legionella* spp. | 12/179 (6.7)** | | 307/2,152 (14.3) |  | |
|  |  |  | *H. influenzae* | 9/179 (5.0) | | 130/2,152 (6.0) |  | |
|  |  |  | *S. aureus* | 8/179 (4.5) | | 81/2,152 (3.8) |  | |
|  |  |  | Virus | 8/179 (4.5) | | 131/2,152 (6.1) |  | |
|  |  |  | *Pseudomonas* spp. | 2/179 (1.1) | | 42/2,152 (2.0) |  | |
|  |  |  | Enterobacteria | 1/179 (0.6)** | | 149/2,152 (6.9) |  | |
|  |  |  | *M. catarrhalis* | 1/179 (0.6) | | 20/2,152 (0.9) |  | |
|  |  |  | *C. pneumoniae* | 0 | | 5/2,152 (0.2) |  | |
|  |  | **Outpatients** |  | **Age <65 yrs**  **(*n*=1,809)** | | **Age ≥65 yrs**  **(*n*=626)** |  | |
|  |  | [****p*<0.001 versus patients ≥65 yrs] |  |  |  |  |  |  |
|  |  |  | Patients with pathogens identified | 574/1,809 (31.7) | | 156/626 (24.9) |  | |
|  |  |  | *M. pneumoniae* | 197/574 (34.3)*** | | 9/156 (5.8) |  | |
|  |  |  | *S. pneumoniae* | 107/574 (18.6) | | 33/156 (21.2) |  | |
|  |  |  | *Legionella* spp. | 59/574 (10.3) | | 27/156 (17.3) |  | |
|  |  |  | *H. influenzae* | 51/574 (8.9) | | 22/156 (14.1) |  | |
|  |  |  | Virus | 43/574 (7.5) | | 10/156 (6.4) |  | |
|  |  |  | *S. aureus* | 14/574 (2.4) | | 8/156 (5.1) |  | |
|  |  |  | Enterobacteria | 9/574 (1.6)*** | | 14/156 (9.0) |  | |
|  |  |  | *M. catarrhalis* | 6/574 (1.0) | | 2/156 (1.3) |  | |
|  |  |  | *C. pneumoniae* | 2/574 (0.3) | | 0 |  | |
|  |  |  | *Pseudomonas* spp. | 1/574 (0.2) | | 1/156 (0.6) |  | |
|  |  | **Outpatients** |  | **Age <50 yrs**  **(*n*=1,149)** | | **Age ≥50 yrs**  **(*n*=1,286)** |  | |
|  |  | [***p*<0.01, ****p*<0.001 versus patients ≥50 yrs] |  |  |  |  |  |  |
|  |  |  | Patients with pathogens identified | 391/1,149 (34.0) | | 339/1,286 (26.4) |  | |
|  |  |  | *M. pneumoniae* | 175/391 (44.8)*** | | 31/339 (9.1) |  | |
|  |  |  | *S. pneumoniae* | 72/391 (18.4) | | 68/339 (20.1) |  | |
|  |  |  | *H. influenzae* | 33/391 (8.4) | | 40/339 (11.8) |  | |
|  |  |  | *Legionella* spp. | 31/391 (7.9)** | | 55/339 (16.2) |  | |
|  |  |  | Virus | 24/391 (6.1) | | 29/339 (8.6) |  | |
|  |  |  | Enterobacteria | 4/391 (1.0)*** | | 19/339 (5.6) |  | |
|  |  |  | *S. aureus* | 3/391 (0.8)*** | | 19/339 (5.6) |  | |
|  |  |  | *C. pneumoniae* | 2/391 (0.5) | | 0 |  | |
|  |  |  | *M. catarrhalis* | 1/391 (0.3) | | 7/339 (2.1) |  | |
|  |  |  | *Pseudomonas* spp. | 0 | | 2/339 (0.6) |  | |
|  |  | **Outpatients** |  | **Age <40 yrs**  **(*n*=682)** | | **Age ≥40 yrs**  **(*n*=1,753)** |  | |
|  |  | [***p*<0.01, ****p*<0.001 versus patients ≥40 yrs] |  |  | |  |  |  |
|  |  |  | Patients with pathogens identified | 239/682 (35.0) | | 491/1,753 (28.0) |  | |
|  |  |  | *M. pneumoniae* | 123/239 (51.5)*** | | 83/491 (16.9) |  | |
|  |  |  | *S. pneumoniae* | 42/239 (17.6) | | 98/491 (20.0) |  | |
|  |  |  | *H. influenzae* | 23/239 (9.6) | | 50/491 (10.2) |  | |
|  |  |  | *Legionella* spp. | 17/239 (7.1)** | | 69/491 (14.1) |  | |
|  |  |  | Virus | 11/239 (4.6) | | 42/491 (8.6) |  | |
|  |  |  | *S. aureus* | 3/239 (1.3) | | 19/491 (3.9) |  | |
|  |  |  | *C. pneumoniae* | 2/239 (0.8) | | 0 |  | |
|  |  |  | Enterobacteria | 2/239 (0.8) | | 21/491 (4.3) |  | |
|  |  |  | *M. catarrhalis* | 1/239 (0.4) | | 7/491 (1.4) |  | |
|  |  |  | *Pseudomonas* spp. | 0 | | 2/491 (0.4) |  | |
|  |  | **Outpatients** |  | **Age <30 yrs**  **(*n*=255)** | | **Age ≥30 yrs**  **(*n*=2,180)** |  | |
|  |  | [****p*<0.001 versus patients ≥30 yrs] |  |  |  |  |  |  |
|  |  |  | Patients with pathogens identified | 93/255 (36.5) | | 637/2,180 (29.2) |  | |
|  |  |  | *M. pneumoniae* | 57/93 (61.3)*** | | 149/637 (23.4) |  | |
|  |  |  | *S. pneumoniae* | 13/93 (14.0) | | 127/637 (19.9) |  | |
|  |  |  | *H. influenzae* | 7/93 (7.5) | | 66/637 (10.4) |  | |
|  |  |  | Virus | 6/93 (6.5) | | 47/637 (7.4) |  | |
|  |  |  | *Legionella* spp. | 5/93 (5.4) | | 81/637 (12.7) |  | |
|  |  |  | *S. aureus* | 2/93 (2.2) | | 20/637 (3.1) |  | |
|  |  |  | Enterobacteria | 1/93 (1.1) | | 22/637 (3.5) |  | |
|  |  |  | *C. pneumoniae* | 0 | | 2/637 (0.3) |  | |
|  |  |  | *M. catarrhalis* | 0 | | 8/637 (1.3) |  | |
|  |  |  | *Pseudomonas* spp. | 0 | | 2/637 (0.3) |  | |
|  |  | **Hospitalised patients** |  | **Age <65 yrs**  **(*n*=2,274)** | | **Age ≥65 yrs**  **(*n*=3,094)** |  | |
|  |  | [***p*<0.01, ****p*<0.001 versus patients ≥65 yrs] |  |  |  |  |  |  |
|  |  |  | Patients with pathogens identified | 764/2,274 (33.6) | | 837/3,094 (27.1) |  | |
|  |  |  | *S. pneumoniae* | 268/764 (35.1) | | 297/837 (35.5) |  | |
|  |  |  | *M. pneumoniae* | 148/764 (19.4)*** | | 36/837 (4.3) |  | |
|  |  |  | *Legionella* spp. | 118/764 (15.4) | | 115/837 (13.7) |  | |
|  |  |  | Enterobacteria | 43/764 (5.6)** | | 84/837 (10.0) |  | |
|  |  |  | *S. aureus* | 37/764 (4.8) | | 30/837 (3.6) |  | |
|  |  |  | *H. influenzae* | 35/764 (4.6) | | 31/837 (3.7) |  | |
|  |  |  | Virus | 18/764 (2.4)*** | | 68/837 (8.1) |  | |
|  |  |  | *Pseudomonas* spp. | 15/764 (2.0) | | 27/837 (3.2) |  | |
|  |  |  | *C. pneumoniae* | 3/764 (0.4) | | 0 |  | |
|  |  |  | *M. catarrhalis* | 3/764 (0.4) | | 10/837 (1.2) |  | |
|  |  | **Hospitalised patients** |  | **Age <50 yrs**  **(*n*=1,144)** | | **Age ≥50 yrs**  **(*n*=4,224)** |  | |
|  |  | [***p*<0.01, ****p*<0.001 versus patients ≥50 yrs] |  |  |  |  |  |  |
|  |  |  | Patients with pathogens identified | 414/1,144 (36.2) | | 1,187/4,224 (28.1) |  | |
|  |  |  | *S. pneumoniae* | 137/414 (33.1) | | 428/1,187 (36.1) |  | |
|  |  |  | *M. pneumoniae* | 124/414 (30.0)*** | | 60/1,187 (5.1) |  | |
|  |  |  | *Legionella* spp. | 58/414 (14.0) | | 175/1,187 (14.7) |  | |
|  |  |  | *S. aureus* | 23/414 (5.6) | | 44/1,187 (3.7) |  | |
|  |  |  | *H. influenzae* | 18/414 (4.3) | | 48/1,187 (4.0) |  | |
|  |  |  | Virus | 9/414 (2.2)** | | 77/1,187 (6.5) |  | |
|  |  |  | Enterobacteria | 8/414 (1.9)*** | | 119/1,187 (10.0) |  | |
|  |  |  | *Pseudomonas* spp. | 6/414 (1.4) | | 36/1,187 (3.0) |  | |
|  |  |  | *C. pneumoniae* | 2/414 (0.5) | | 1/1,187 (0.1) |  | |
|  |  |  | *M. catarrhalis* | 2/414 (0.5) | | 11/1,187 (0.9) |  | |
|  |  | **Hospitalised patients** |  | **Age <40 yrs**  **(*n*=656)** | | **Age ≥40 yrs**  **(*n*=4,712)** |  | |
|  |  | [***p*<0.01, ****p*<0.001 versus patients ≥40 yrs] |  |  |  |  |  |  |
|  |  |  | Patients with pathogens identified | 238/656 (36.3) | | 1,363/4,712 (28.9) |  | |
|  |  |  | *M. pneumoniae* | 88/238 (37.0)*** | | 96/1,363 (7.0) |  | |
|  |  |  | *S. pneumoniae* | 76/238 (31.9) | | 489/1,363 (35.9) |  | |
|  |  |  | *Legionella* spp. | 26/238 (10.9) | | 207/1,363 (15.2) |  | |
|  |  |  | *S. aureus* | 14/238 (5.9) | | 53/1,363 (3.9) |  | |
|  |  |  | *H. influenzae* | 10/238 (4.2) | | 56/1,363 (4.1) |  | |
|  |  |  | Enterobacteria | 6/238 (2.5)** | | 121/1,363 (8.9) |  | |
|  |  |  | Virus | 6/238 (2.5) | | 80/1,363 (5.9) |  | |
|  |  |  | *Pseudomonas* spp. | 3/238 (1.3) | | 39/1,363 (2.9) |  | |
|  |  |  | *C. pneumoniae* | 2/238 (0.8) | | 1/1,363 (0.1) |  | |
|  |  |  | *M. catarrhalis* | 1/238 (0.4) | | 12/1,363 (0.9) |  | |
|  |  | **Hospitalised patients** |  | **Age <30 yrs**  **(*n*=248)** | | **Age ≥30 yrs**  **(*n*=5,120)** |  | |
|  |  | ****p*<0.001 versus patients ≥30 yrs] |  |  |  |  |  |  |
|  |  |  | Patients with pathogens identified | 86/248 (34.7) | | 1,515/5,120 (29.6) |  | |
|  |  |  | *M. pneumoniae* | 40/86 (46.5)*** | | 144/1,515 (9.5) |  | |
|  |  |  | *S. pneumoniae* | 22/86 (25.6) | | 543/1,515 (35.8) |  | |
|  |  |  | *Legionella* spp. | 7/86 (8.1) | | 226/1,515 (14.9) |  | |
|  |  |  | *S. aureus* | 6/86 (7.0) | | 61/1,515 (4.0) |  | |
|  |  |  | *H. influenzae* | 2/86 (2.3) | | 64/1,515 (4.2) |  | |
|  |  |  | *Pseudomonas* spp. | 2/86 (2.3) | | 40/1,515 (2.6) |  | |
|  |  |  | Virus | 2/86 (2.3) | | 84/1,515 (5.5) |  | |
|  |  |  | *M. catarrhalis* | 1/86 (1.2) | | 12/1,515 (0.8) |  | |
|  |  |  | *C. pneumoniae* | 0 | | 3/1,515 (0.2) |  | |
|  |  |  | Enterobacteria | 0 | | 127/1,515 (8.4) |  | |
| Kothe et al. 2008 [23] | - Culture of sputum, blood, pleural fluid, transthoracic needle aspiration, BAS, protected specimen brush and BAL fluid samples - Urine antigen tests for *S. pneumoniae* and *Legionella* | Patients with CAP |  | **Age <65 yrs**  **(*n*=1,298)** | | **Age ≥65 yrs**  **(*n*=1,349)** |  | |
|  |  | [****p*<0.001 versus patients ≥65 yrs] |  |  |  |  |  |  |
|  |  |  | Patients with pathogens identified | 271/1,298 (20.9) | | 268/1,349 (19.9) |  | |
|  |  |  | *S. pneumoniae* | 114/271 (42.1) | | 116/268 (43.3) |  | |
|  |  |  | *Legionella* spp. | 45/271 (16.6) | | 47/268 (17.5) |  | |
|  |  |  | *M. pneumoniae* | 38/271 (14.0)*** | | 2/268 (0.7) |  | |
|  |  |  | Virus | 21/271 (7.7) | | 50/268 (18.6) |  | |
|  |  |  | *H. influenzae* | 13/271 (4.8) | | 9/268 (3.4) |  | |
|  |  |  | Gram-negative bacilli | 10/271 (3.7) | | 19/268 (7.1) |  | |
|  |  |  | *S. aureus* | 4/271 (1.5) | | 6/268 (2.2) |  | |
|  |  |  | *C. pneumoniae* | 3/271 (1.1) | | 0 |  | |
| Le Moing et al. 2006 [35] | - Culture of blood or a clinically relevant respiratory specimen - Serology | HIV patients on protease inhibitor therapy, hospitalised with bacterial pneumonia (*n*=29) | 11 episodes of definite bacterial pneumonia |  | |  |  | |
|  |  |  | *S. pneumoniae* | 9/11 (81.8) | |  |  | |
|  |  |  | *H. influenzae* | 1/11 (9.1) | |  |  | |
|  |  |  | *L. pneumophila* | 1/11 (9.1) | |  |  | |
| Liapikou et al. 2012 [42] | - Blood cultures - Urinary antigen tests for *S. pneumoniae* and *L. pneumophila* - Sputum in all patients when possible - BAS in all intubated patients - Pleural fluid by thoracocentesis - Paired serology when possible to detect *C. pneumoniae* and *L. pneumophila*, *C. burnetii* and respiratory viruses (i.e. influenza viruses A and B, parainfluenza viruses 1–3, RSV and adenovirus) | Patients hospitalised with CAP  [**p*<0.05, ***p*<0.01 versus no COPD] |  | **No COPD**  **(*n*=1,167)** | | **With COPD**  **(*n*=212)** |  | |
|  |  |  | Patients with pathogens identified | 537/1,167 (46) | | 95/212 (44.8) |  | |
|  |  |  | *S. pneumoniae* | 230/537 (42.8) | | 36/95 (37.9) |  | |
|  |  |  | Virus | 67/537 (12.5) | | 13/95 (13.7) |  | |
|  |  |  | *L. pneumophila* | 42/537 (7.8) | | 2/95 (2.1)* |  | |
|  |  |  | *C. pneumoniae* | 22/537 (4.1) | | 2/95 (2.1) |  | |
|  |  |  | *M. pneumoniae* | 18/537 (3.4) | | 2/95 (2.1) |  | |
|  |  |  | *S. aureus* | 17/537 (3.2) | | 0 |  | |
|  |  |  | *H. influenzae* | 12/537 (2.2) | | 1/95 (1.1) |  | |
|  |  |  | *E. coli* | 7/537 (1.3) | | 2/95 (2.1) |  | |
|  |  |  | *P. aeruginosa* | 5/537 (0.9) | | 7/95 (7.4)** |  | |
|  |  |  | *K. pneumoniae* | 5/537 (0.9) | | 0 |  | |
|  |  |  | *M. catarrhalis* | 2/537 (0.4) | | 0 |  | |
| Maddedu et al. 2008 [36] | - Cultures of sputum and blood samples - Urinary antigen tests for *S. pneumoniae* and *L. pneumophila* - Serology for *Chlamydia* species and *M. pneumoniae* | HIV patients hospitalised for CAP | Episodes with pathogens identified | 30/84 (35.7) | |  |  | |
|  |  | (n=76; 84 episodes) | *S. pneumoniae* | 18/30 (60) | |  |  | |
|  |  |  | *E. coli* | 2/30 (6.7) | |  |  | |
|  |  |  | *H. influenzae* | 2/30 (6.7) | |  |  | |
|  |  |  | *P. aeruginosa* | 2/30 (6.7) | |  |  | |
|  |  |  | *E. faecalis* | 1/30 (3.3) | |  |  | |
|  |  |  | *K. pneumoniae* | 1/30 (3.3) | |  |  | |
|  |  |  | *L. pneumophila* | 1/30 (3.3) | |  |  | |
|  |  |  | *S. aureus* | 1/30 (3.3) | |  |  | |
|  |  |  | *S. marcescens* | 1/30 (3.3) | |  |  | |
|  |  |  | *S. viridans* | 1/30 (3.3) | |  |  | |
| Manno et al. 2009 [37] | - Culture of sputum/BAL or blood | HIV patients hospitalised for CAP |  | **No cirrhosis**  **(*n*=73)** | | **With cirrhosis**  **(*n*=29)** |  | |
|  |  |  | Patients with pathogens identified | 14/73 (19.2) | | 7/29 (24.1) |  | |
|  |  |  | *S. pneumoniae* | 10/14 (71.4) | | 3/7 (42.9) |  | |
|  |  |  | *H. influenzae* | 2/14 (14.3) | | 0 |  | |
|  |  |  | *Enterobacteriaceae* | 1/14 (7.1) | | 3/7 (42.9) |  | |
| Molinos et al. 2009 [38] | - Two sets of blood cultures - Sputum culture (on hospital admission and prior to antibiotic administration) - Pleural fluid culture - Serology (collected first day and 4–6 weeks thereafter) for *M. pneumoniae*, *C. psittaci*, *C. pneumoniae*, *L. pneumophila*, *C. burnetii*, influenza virus types A and B, parainfluenzavirus types 1–3, adenovirus and RSV - Urinary antigen detection of *L. pneumophila* and *S. pneumoniae* - BAS and bronchoscopic samples obtained according to clinical judgement | Patients hospitalised with CAP |  | **No COPD**  **(*n*=466)** | | **With COPD**  **(*n*=244)** |  | |
|  |  |  | Patients with pathogens identified | 179/466 (38.0) | | 95/244 (39.0) |  | |
|  |  |  | *S. pneumoniae* | 102/179 (57.0) | | 63/95 (66.3) |  | |
|  |  |  | *L. pneumophila* | 36/179 (20.1) | | 4/95 (4.2) |  | |
|  |  |  | *M. pneumoniae* | 9/179 (5.0) | | 3/95 (3.2) |  | |
|  |  |  | *C. pneumoniae* | 8/179 (4.5) | | 6/95 (6.3) |  | |
|  |  |  | *C. burnetii* | 6/179 (3.4) | | 2/95 (2.1) |  | |
|  |  |  | Virus | 5/179 (2.8) | | 5/95 (5.3) |  | |
|  |  |  | *H. influenzae* | 3/179 (1.7) | | 1/95 (1.1) |  | |
|  |  |  | *E. coli* | 2/179 (1.1) | | 1/95 (1.1) |  | |
|  |  |  | *P. aeruginosa* | 0 | | 2/95 (2.1) |  | |
|  |  |  | *K. pneumoniae* | 0 | | 1/95 (1.1) |  | |
|  |  |  | *S. aureus* | 0 | | 1/95 (1.1) |  | |
|  |  |  | *S. mitis* | 0 | | 1/95 (1.1) |  | |
| Pérez-Sola et al. 2011 [67] | Not reported | Patients with rheumatic diseases treated with TNF antagonists and diagnosed with pneumonia (*n*=101) | Patients with pathogens identified | 25/101 (24.0) | |  |  | |
|  |  |  | *Legionella* spp. | 5/25 (20.0) | |  |  | |
|  |  |  | *S. aureus* | 5/25 (20.0) | |  |  | |
|  |  |  | *S. pneumoniae* | 3/25 (12.0) | |  |  | |
|  |  |  | *P. aeruginosa* | 2/25 (8.0) | |  |  | |
| Ruiz et al. 2010 [31] | - Urinary antigen test for *S. pneumoniae* and *L. pneumophila* - Blood culture (patients with body temperature 37.5°C and/or suspected of having a LRTI | Patients hospitalised with bacteraemic CAP due to Gram-negative bacteria (*n*=51) | Patients with pathogens identified | 51/51 (100) | |  |  | |
|  |  |  | *E. coli* | 30/51 (58.8) | |  |  | |
|  |  |  | *K. pneumoniae* | 9/51 (17.6) | |  |  | |
|  |  |  | *H. influenzae* | 5/51 (9.8) | |  |  | |
|  |  |  | *P. aeruginosa* | 3/51 (5.9) | |  |  | |
|  |  |  | *A. baumannii* | 1/51 (2.0) | |  |  | |
|  |  |  | *E. sakazakii* | 1/51 (2.0) | |  |  | |
|  |  |  | *P. mirabilis* | 1/51 (2.0) | |  |  | |
|  |  |  | *S. maltophilia* | 1/51 (2.0) | |  |  | |
| Viasus et al. 2011 [50] | - Blood, pleural effusion, sputum and other samples - Urinary antigen test for *S. pneumoniae* and *L. pneumophila* - Serology to determine antibodies against atypical agents (on admission and 3–4 weeks thereafter) | Patients hospitalised with CAP |  | **No kidney disease**  **(*n*=3,597)** | | **Chronic kidney disease**  **(*n*=203)** |  | |
|  |  |  | Patients with pathogens identified | 2,178/3,597 (60.6) | | 103/203 (50.7) |  | |
|  |  |  | *S. pneumoniae* | 1,248/2,178 (57.3) | | 57/103 (55.3) |  | |
|  |  |  | Atypical agents | 225/2,178 (10.3) | | 7/103 (6.8) |  | |
|  |  |  | *L. pneumophila* | 204/2,178 (9.4) | | 9/103 (8.7) |  | |
|  |  |  | *H. influenzae* | 184/2,178 (8.4) | | 14/103 (13.6) |  | |
|  |  |  | Gram-negative bacilli | 67/2,178 (3.1) | | 3/103 (2.9) |  | |
|  |  |  | *S. aureus* | 18/2,178 (0.8) | | 1/103 (1.0) |  | |
| Viasus et al. 2011 [46] | - Blood, pleural effusion, sputum and other samples - Urinary antigen test for *S. pneumoniae* and *L. pneumophila* - Serology to determine antibodies against atypical agents (on admission and 3–4 weeks thereafter) | Patients hospitalised with CAP  [***p*<0.01 versus no cirrhosis] |  | **No cirrhosis**  **(*n*=3,330)** | | **Cirrhosis**  **(*n*=90)** |  | |
|  |  |  | Patients with pathogens identified | 1,952/3,330 (58.6) | | 66/90 (73.3)** |  | |
|  |  |  | *S. pneumoniae* | 1,099/1,952 (56.3) | | 42/66 (63.)** |  | |
|  |  |  | *L. pneumophila* | 203/1,952 (10.4) | | 4/66 (6.1) |  | |
|  |  |  | *H. influenzae* | 186/1,952 (9.5) | | 6/66 (9.1) |  | |
|  |  |  | Gram-negative bacilli | 56/1,952 (2.9) | | 4/66 (6.1) |  | |
|  |  |  | Virus | 36/1,952 (1.8) | | 0 |  | |
|  |  |  | *M. catarrhalis* | 30/1,952 (1.5) | | 1/66 (1.5) |  | |
|  |  |  | *S. aureus* | 16/1,952 (0.8) | | 3/66 (4.5) |  | |
|  |  |  | *N. meningitides* | 4/1,952 (0.2) | | 0 |  | |
|  |  |  | *A. fumigatus* | 0 | | 1/66 (1.5) |  | |
| Vila-Corcoles et al. 2009 [26] | - Blood cultures - Sputum cultures - Paired serology (at an interval of 3–8 weeks) - Urinary antigen tests for *S. pneumoniae* and *Legionella* | Patients ≥65 yrs with CAP (*n*=473)  142 pathogens detected in 131 patients (10 cases of CAP caused by mixed microorganisms) | Patients with pathogens identified | 131/473^†^ (27.7) | |  |  | |
|  |  |  | *S. pneumoniae* | 70/131 (53.4) | |  |  | |
|  |  |  | *P. aeruginosa* | 22/131 (16.8) | |  |  | |
|  |  |  | *C. pneumoniae* | 13/131 (9.9) | |  |  | |
|  |  |  | *H. influenzae* | 8/131 (6.1) | |  |  | |
|  |  |  | *S. aureus* | 7/131 (5.3) | |  |  | |
|  |  |  | *Legionella* | 4/131 (3.1) | |  |  | |
|  |  |  | *Acinetobacter* | 3/131 (2.3) | |  |  | |
|  |  |  | *M. catharralis* | 3/131 (2.3) | |  |  | |
|  |  |  | *S. marcescens* | 3/131 (2.3) | |  |  | |
|  |  |  | *K. pneumoniae* | 2/131 (1.5) | |  |  | |
|  |  |  | *C. burnetii* | 1/131 (0.8) | |  |  | |
|  |  |  | *M. tuberculosis* | 1/131 (0.8) | |  |  | |
|  |  |  | *N. asteroides* | 1/131 (0.8) | |  |  | |
|  |  |  | *P. mirabillis* | 1/131 (0.8) | |  |  | |
|  |  |  | *Peptococcus* | 1/131 (0.8) | |  |  | |
|  |  |  | *S. salivaris* | 1/131 (0.8) | |  |  | |
|  |  |  | *S. sanguis* | 1/131 (0.8) | |  |  | |

*A. baumannii, Acinetobacter baumannii; A. hydrophila, Aeromonas hydrophila; A. fumigatus, Aspergillus fumigatus; C. trachomatis, Chlamydia trachomatis; C. pneumoniae, Chlamydophila pneumoniae; C. psittaci, Chlamydophila psittaci; C. freundii, Citrobacter freundii; C. Burnetii, Coxiella burnetii; E. sakazakii, Enterobacter sakazakii; E. faecalis, Enterococcus faecalis; E. coli, Escherichia coli; H. influenzae, Haemophilus influenzae; K. oxytoca, Klebsiella oxytoca; K. pneumoniae, Klebsiella pneumoniae; L. pneumophila, Legionella pneumophila; M. catarrhalis, Moraxella catarrhalis; M. tuberculosis, Mycobacterium tuberculosis; M. pneumoniae, Mycoplasma pneumoniae; N. asteroids, Nocardia asteroides; N. meningitides, Neisseria meningitides; P. mirabilis, Proteus mirabilis; P. aeruginosa, Pseudomonas aeruginosa; S. marcescens, Serratia marcescens; S. aureus, Staphylococcus aureus; S. maltophilia, Stenotrophomonas maltophilia; S. mitis, Streptococcus mitis; S. pneumoniae, Streptococcus pneumoniae; S. pyogenes; Streptococcus pyogenes; S. salivaris, Streptococcus salivaris; S. sanguis, Streptococcus sanguis; S. viridans, Streptococcus viridians*

BAL, bronchoalveolar lavage; BAS, tracheobronchial aspirates; CAP, community-acquired pneumonia; COPD, chronic obstructive pulmonary disease; HIV, human immunodeficiency virus; LRTI, lower respiratory tract infection; MRSA, methicillin-resistant *S. aureus*; MSSA, methicillin-susceptible *S. aureus*; N/A, not applicable; PPI, proton pump inhibitor; PSI, pneumonia severity index score; spp., species; RSV, respiratory syncytial virus; TNF, tumour necrosis factor; yr/yrs, year/years

**^†^**Aetiological examination was carried out in only 358 cases
