# Supplementary material for: The aetiology and antibiotic management of community-acquired pneumonia in adults in Europe: a literature review
Source: Eur J Clin Microbiol Infect Dis. 2014 Feb 15;33(7):1065–79. doi: 10.1007/s10096-014-2067-1 (PMC4042014; doi:10.1007/s10096-014-2067-1)
Supplement: Supplementary file 2 — (DOC 443 kb) [file 10096_2014_2067_MOESM2_ESM.doc]

**Supplementary table 2.** Antibiotic use in patients with CAP.

| **Citation** | **Population** | **Age (yrs)** | **Antibiotic use** | | | **Inappropriate versus appropriate antibiotic therapy** | | **Antibiotic resistance** | **Outcomes of antibiotic treatment** |
| --- | --- | --- | --- | --- | --- | --- | --- | --- | --- |
| Cabre 2010 [25] | Patients ≥70 years with CAP requiring hospitalization, n=134 (32% nursing home residents) | Mean 84.51±6.8 | According to hospital guidelines:   - Amoxicillin 1 g i.v./8 h - Severe pneumonia: ceftriaxone 2 g i.v./24 h and clarithromycin 500 mg i.v. or oral/12 h - For patients allergic to penicillin: levofloxacin 500 mg/day - If aspiration pneumonia suspected: amoxicillin–clavulanic acid, 2 g i.v./8 h | | | Not reported | | Not reported | Not reported |
| Carratalà 2007 [43] | Patients with CAP requiring hospitalization (n=601) | Mean 63.7±17.1 | Monotherapy (408/601, 67.9%):   - β-Lactams (252/601, 41.9%) - Quinolones (152/601, 25.3%) - Other (4/601, 0.7%)   Combination therapy (193/601, 32.1%):   - β-Lactams + quinolones (169/601, 28.1%) - β-Lactams + macrolides (10/601, 1.7%) - Other combinations (14/601, 2.3%) | | | Inappropriate antibiotic therapy: (12/601, 2.0%) | | Patients with resistant pneumococcal strains to:   - Penicillin 14.9% - Ceftriaxone 3.5% - Erythromycin 15.8%   Patients with resistant *H. influenzae* strains:   - β-lactamase production in 9.7% of isolates | Not reported |
| Chidiac 2012 [27] | Hospitalized patients with community-acquired Legionnaires’ disease (n=540) | Median 60 (range 17–100) | Not reported | | | Appropriate empirical antibiotic treatment before hospital admission: 25/174 (14.4%)  Appropriate antibiotics upon admission (empirically): 292/412 (70.8%)  Appropriate antibiotics upon admission (empirically) and after confirmation of diagnosis: 537/538 (99.8%) | | Not reported | Not reported |
| Cillóniz 2011 [22] | Patients with CAP admitted to ICU (n=362) | Mean 63.4±16.5 | Data available in 96% (n=347) patients  Most frequent regimens:   - Fluoroquinolones plus β-lactam (217/347, 63%) - β-Lactam plus macrolide (73/347, 21%) - Fluoroquinolone monotherapy (39/347, 11%) - β-Lactam monotherapy (18/347, 5%) | | | Inappropriate empirical treatment:   - Monomicrobial CAP (15/157, 10%) - Polymicrobial CAP (15/39, 39%, p<0.001)   Excluding respiratory viruses, pathogens most frequently associated with inadequate treatment:   - MRSA (10 cases) - *S. pneumoniae* (9 cases) - *P. aeruginosa* (9 cases) - Gram-negative enteric bacilli (9 cases) | | Not reported | Strong association between polymicrobial aetiology and initial inappropriate antimicrobial treatment, which in turn was an independent predictor of increased hospital mortality.  Inappropriate empiric treatment:   - Univariate analysis: OR 11.23 (95% CI 4.44–28.38), p<0.001 - Multivariate analysis: adjusted OR 10.79 (3.97-29.30), p<0.001 |
| Cillóniz 2012 [44] | Patients hospitalized with pneumococcal pneumonia (n=626) | Mean 63.6±18.9  (46% ≤65 years) | Data available for 620/626 patients   - β-Lactam plus macrolide (241/620, 39%) - β-Lactam plus fluoroquinolone (168/620, 27%) - Fluoroquinolone alone (136/620, 22%) - β-Lactam alone (52/620, 8%) - Macrolide plus fluoroquinolone (6/620, 1%) - Macrolide alone (2/620, 0.3%) - Other combinations (15/620, 2%)   Complicated CAP (n=235):   - β-Lactam plus macrolide (90/235, 38.3%) - β-Lactam plus fluoroquinolone (85/235, 36.2%) - Fluoroquinolone alone (32/235, 13.6%) - β-Lactam alone (22/235, 9.4%) - Macrolide plus fluoroquinolone (1/235, 0.4%) - Macrolide alone (0) - Other combinations (4/235, 1.7%)   Uncomplicated CAP (n=391):   - β-Lactam plus macrolide (151/391, 38.6%) - β-Lactam plus fluoroquinolone (83/391, 21.2%, p<0.001 versus complicated CAP) - Fluoroquinolone alone (104/391, 26.6%, p<0.001 versus complicated CAP) - β-Lactam alone (30/391, 7.7%) - Macrolide plus fluoroquinolone (5/391, 1.3%) - Macrolide alone (2/391, 0.5%) - Other combinations (11/391, 2.8%) | | | No cases of inadequate antibiotic treatment | | Pneumococcal isolates resistant to penicillin (69/333, 20.7%):   - Intermediate (MIC 4 mg/L) (38/69) - High (MIC ≥8 mg/L) (31/69)   Pneumococcal isolates resistant to erythromycin (56/328, 17.1%):   - Intermediate (MIC 0.5 mg/L) (2/56) - High (MIC ≥1 mg/L) (54/56)   Penicillin and erythromycin resistance was almost twofold higher in uncomplicated CAP cases (p=0.036 and, p=0.027, respectively) | Not reported |
| de Roux 2006 [45] | Patients hospitalized for CAP, classified according to alcohol abuse status:  Current, n=128  Former, n=54  None, n=1165 | Current alcohol abuse, 58±14 | Most common regimen:  Cephalosporin combined with macrolide:   - Current abuse (70%) - Former abuse (67%) - No abuse (67%) | | | Not reported | | Patients with intermediate pneumococcal resistance to penicillin:   - No abuse (16/187, 9%) - Current abuse (3/34, 9%) - Former abuse (2/16, 12%)   Patients with high level pneumococcal resistance to penicillin:   - No abuse (32/187, 17%) - Current abuse (5/34, 15%) - Former abuse (1/16, 6%)   Patients with pneumococcal resistance to erythromycin:   - No abuse (39/187, 21%) - Current abuse (6/34, 18%) - Former abuse (2/16, 12%) | Not reported |
|  |  | Former alcohol abuse, 71±11 |  |  |  |  |  |  |  |
|  |  | No alcohol abuse, 68±19 |  |  |  |  |  |  |  |
| Garcia-Vidal 2009 [48] | Patients hospitalized with CAP (n=1556)  Recurrent CAP (≥2 episodes of CAP in 3 yrs with asymptomatic period ≥1 month) (n=146)  Non-recurrent CAP (n=1410) | Mean:  Recurrent CAP 70.96±13.824  Non-recurrent CAP 65.03±16.573 | According to hospital guidelines:   - β-Lactam (ceftriaxone or amoxicillin-clavulanate) with or without a macrolide or fluoroquinolone - Combination therapy (patients with clinical suspicion of *Legionella* or an atypical pathogen, or in the absence of a demonstrative sputum Gram stain) - Levofloxacin monotherapy allowed for selected cases | | | Percentage of patients receiving discordant empirical antibiotic therapy was similar in both groups (approximately 5%) | | Not reported | Not reported |
| Giannella 2012 [49] | Adults (≥16 yrs) treated for CAP in the internal medicine department (n=591) | Median 77 (IQR 65–84) | - Empirical therapy (588/591, 99.5%) - Adherence to IDSA 2007 guidelines (413/591, 69.9%) | | | Adequacy of empirical therapy (54/68, 79.4%) | | Not reported | Not reported |
| Gutierrez 2005 [40] | Adults (≥15 yrs) with CAP (n=493)  (n=490 with treatment information available) | Mean 56.6 (range 15–94) | Combined therapy :   - Macrolide + β-lactam (223/490, 45.5%) - β-Lactam monotherapy (106/490, 21.6%) - Macrolide monotherapy (46/490, 9.4%) - Fluoroquinolone (85/490, 17.3%) - Other antibiotics (30/490, 6.1%)   Antibiotics prescribed varied according to site of care:   - Monotherapy with macrolides were prescribed predominantly in the outpatient setting (30/46, 65.2%) prescriptions - Combined therapy was given mostly to patients admitted to hospital (191/223, 85.7% prescriptions) - Fluoroquinolones (43/85, 50.6% courses administered to outpatients and 42/85 49.4% to hospitalized patients) | | | Not reported | | Not reported | No association between combined antimicrobial therapy and either complications or mortality in patients with any particular aetiology of CAP  Combined antimicrobial therapy including either a macrolide or a fluoroquinolone was associated with reduced mortality by univariate analysis:   Antibiotic therapy not including a macrolide or fluoroquinolone:   - univariate analysis: OR 2.75 (95% CI 1.21–6.29), p=0.01   The association was not confirmed by multivariate analysis |
| Klapdor 2012 [41] | Adults (≥18 yrs) with CAP (n=7803)  <65 yrs, n=4083 (2.6% nursing home residents)  ≥65 yrs, n=3270 (14.4% nursing home residents) | Overall: mean 60.9±18.5 (range 18–101)  <65 yrs: median 47.0 (IQR 20.7)  ≥65 yrs: median 76.0 (IQR 11.8) | Total population [***p<0.001] | **Age <65 yrs (n=4083)** | **Age ≥65 yrs (n=3720)** | | Not reported | Not reported | Not reported |
|  |  |  | Monotherapy | 2856/3996 (71.5%)*** | 2450/3680 (66.6%) | |  |  |  |
|  |  |  | Combination therapy | 1120/3996 (28.0%)*** | 1221/3680 (33.2%) | |  |  |  |
|  |  |  | β-Lactams | 2499/3996 (62.5%)*** | 2990/3680 (81.3%) | |  |  |  |
|  |  |  | Macrolides | 1302/3996 (32.6%) | 1155/3680 (31.4%) | |  |  |  |
|  |  |  | Quinolones | 1128/3996 (28.2%)*** | 631/3680 (17.1%) | |  |  |  |
|  |  |  | Tetracylines | 38/3996 (1.0%) | 22/3680 (0.6%) | |  |  |  |
|  |  |  | Glycopeptides | 4/3996 (0.1%) | 6/3680 (0.2%) | |  |  |  |
|  |  |  | Lincosamides | 20/3996 (0.5%) | 21/3680 (0.6%) | |  |  |  |
|  |  |  | Ketolides | 69/3996 (1.7%)*** | 23/3680 (0.6%) | |  |  |  |
|  |  |  | Co-trimoxazole | 6/3996 (0.2%) | 10/3680 (0.3%) | |  |  |  |
|  |  |  | Aminoglycosides | 19/3996 (0.5%) | 12/3680 (0.3%) | |  |  |  |
|  |  |  | Other | 21/3996 (0.5%) | 22/3680 (0.6%) | |  |  |  |
|  |  |  | **Total population [***p<0.001]** | **Age <50 yrs (n=2293)** | **Age ≥50 yrs (n=5510)** | | Not reported | Not reported | Not reported |
|  |  |  | Monotherapy | 1618/2242 (72.2%)*** | 3688/5434 (67.9%) | |  |  |  |
|  |  |  | Combination therapy | 611/2242 (27.3%)*** | 1730/5434 (31.8%) | |  |  |  |
|  |  |  | β-Lactams | 1342/2242 (59.9%)*** | 4147/5434 (76.3%) | |  |  |  |
|  |  |  | Macrolides | 737/2242 (32.9%) | 1720/5434 (31.7%) | |  |  |  |
|  |  |  | Quinolones | 641/2242 (28.6%)*** | 1118/5434 (20.6%) | |  |  |  |
|  |  |  | Tetracylines | 34/2242 (1.5%)*** | 26/5434 (0.5%) | |  |  |  |
|  |  |  | Glycopeptides | 4/2242 (0.2%) | 6/5434 (0.1%) | |  |  |  |
|  |  |  | Lincosamides | 13/2242 (0.6%) | 28/5434 (0.5%) | |  |  |  |
|  |  |  | Ketolides | 47/2242 (2.1%)*** | 45/5434 (0.8%) | |  |  |  |
|  |  |  | Co-trimoxazole | 4/2242 (0.2%) | 12/5434 (0.2%) | |  |  |  |
|  |  |  | Aminoglycosides | 7/2242 (0.3%) | 24/5434 (0.4%) | |  |  |  |
|  |  |  | Other | 15/2242 (0.7%) | 28/5434 (0.5%) | |  |  |  |
|  |  |  | **Total population [***p<0.001, **p<0.01]** | **Age <40 yrs (n=1338)** | **Age ≥40 yrs (n=6465)** | | Not reported | Not reported | Not reported |
|  |  |  | Monotherapy | 943/1309 (72.0%) | 4363/6367 (68.5%) | |  |  |  |
|  |  |  | Combination therapy | 356/1309 (27.2%)** | 1985/6367 (31.2%) | |  |  |  |
|  |  |  | β-Lactams | 773/1309 (59.1%)*** | 4716/6367 (74.1%) | |  |  |  |
|  |  |  | Macrolides | 451/1309 (34.5%) | 2006/6367 (31.5%) | |  |  |  |
|  |  |  | Quinolones | 358/1309 (27.3%)*** | 1401/6367 (22.0%) | |  |  |  |
|  |  |  | Tetracylines | 22/1309 (1.7%)*** | 38/6367  (0.6%) | |  |  |  |
|  |  |  | Glycopeptides | 1/1309 (0.1%) | 9/6367 (0.1%) | |  |  |  |
|  |  |  | Lincosamides | 5/1309 (0.4%) | 36/6367 (0.6%) | |  |  |  |
|  |  |  | Ketolides | 33/1309 (2.5%)*** | 59/6367 (0.9%) | |  |  |  |
|  |  |  | Co-trimoxazole | 2/1309 (0.2%) | 14/6367 (0.2%) | |  |  |  |
|  |  |  | Aminoglycosides | 5/1309 (0.4%) | 26/6367 (0.4%) | |  |  |  |
|  |  |  | Other | 5/1309 (0.4%) | 38/6367 (0.6%) | |  |  |  |
|  |  |  | **Total population [***p<0.001]** | **Age <30 yrs (n=503)** | **Age ≥30 yrs (n=7300)** | | Not reported | Not reported | Not reported |
|  |  |  | Monotherapy | 351/490 (71.6%) | 4955/7186 (69.0%) | |  |  |  |
|  |  |  | Combination therapy | 136/490 (27.8%) | 2205/7186 (30.7%) | |  |  |  |
|  |  |  | β-Lactams | 302/490 (61.6%)*** | 5187/7186 (72.2%) | |  |  |  |
|  |  |  | Macrolides | 169/490 (34.5%) | 2288/7186 (31.8%) | |  |  |  |
|  |  |  | Quinolones | 121/490 (24.7%) | 1638/7186 (22.8%) | |  |  |  |
|  |  |  | Tetracylines | 7/490 (1.4%) | 53/7186 (0.7%) | |  |  |  |
|  |  |  | Glycopeptides | 0 | 10/7186 (0.1%) | |  |  |  |
|  |  |  | Lincosamides | 3/490 (0.6%) | 38/7186 (0.5%) | |  |  |  |
|  |  |  | Ketolides | 17/490 (3.5%)*** | 75/7186 (1.0%) | |  |  |  |
|  |  |  | Co-trimoxazole | 1/490 (0.2%) | 15/7186 (0.2%) | |  |  |  |
|  |  |  | Aminoglycosides | 2/490  (0.4%) | 29/7186  (0.4%) | |  |  |  |
|  |  |  | Other | 0 | 43/7186 (0.6%) | |  |  |  |
|  |  |  | **Outpatients [***p<0.001, **p<0.01]** | **Age <65 yrs (n=1809)** | **Age ≥65 yrs (n=626)** | | Not reported | Not reported | Not reported |
|  |  |  | Monotherapy | 1644/1757 (93.6%) | 575/610 (94.3%) | |  |  |  |
|  |  |  | Combination therapy | 106/1757 (6.0%) | 33/610 (5.4%) | |  |  |  |
|  |  |  | β-Lactams | 737/1757 (41.9%)** | 298/610 (48.9%) | |  |  |  |
|  |  |  | Macrolides | 354/1757 (20.1%)** | 86/610 (14.1%) | |  |  |  |
|  |  |  | Quinolones | 665/1757 (37.8%) | 218/610 (35.7%) | |  |  |  |
|  |  |  | Tetracylines | 26/1757 (1.5%) | 13/610 (2.1%) | |  |  |  |
|  |  |  | Glycopeptides | 1/1757 (0.1%) | 0 | |  |  |  |
|  |  |  | Lincosamides | 5/1757 (0.3%) | 2/610 (0.3%) | |  |  |  |
|  |  |  | Ketolides | 61/1757 (3.5%) | 22/610 (3.6%) | |  |  |  |
|  |  |  | Co-trimoxazole | 1/1757 (0.1%) | 2/610 (0.3%) | |  |  |  |
|  |  |  | Aminoglycosides | 0 | 0 | |  |  |  |
|  |  |  | **Outpatients [**p<0.01]** | **Age <50 yrs (n=1149)** | **Age ≥50 yrs (n=1286)** | | Not reported | Not reported | Not reported |
|  |  |  | Monotherapy | 1036/1118 (92.7%) | 1183/1249 (94.7%) | |  |  |  |
|  |  |  | Combination therapy | 77/1118  (6.9%) | 62/1249  (5.0%) | |  |  |  |
|  |  |  | β-Lactams | 488/1118 (43.6%) | 547/1249 (43.8%) | |  |  |  |
|  |  |  | Macrolides | 236/1118 (21.1%)** | 204/1249 (16.3%) | |  |  |  |
|  |  |  | Quinolones | 393/1118 (35.2%) | 490/1249 (39.2%) | |  |  |  |
|  |  |  | Tetracylines | 22/1118 (2.0%) | 17/1249 (1.4%) | |  |  |  |
|  |  |  | Glycopeptides | 1/1118 (0.1%) | 0 | |  |  |  |
|  |  |  | Lincosamides | 3/1118 (0.3%) | 4/1249 (0.3%) | |  |  |  |
|  |  |  | Ketolides | 42/1118 (3.8%) | 41/1249 (3.3%) | |  |  |  |
|  |  |  | Co-trimoxazole | 1/1118 (0.1%) | 2/1249 (0.2%) | |  |  |  |
|  |  |  | Aminoglycosides | 0 | 0 | |  |  |  |
|  |  |  | **Outpatients** | **Age <40 yrs (n=682)** | **Age ≥40 yrs (n=1753)** | | Not reported | Not reported | Not reported |
|  |  |  | Monotherapy | 620/663 (93.5%) | 1599/1704 (93.8%) | |  |  |  |
|  |  |  | Combination therapy | 39/663 (5.9%) | 100/1704 (5.9%) | |  |  |  |
|  |  |  | β-Lactams | 285/663 (43.0%) | 750/1704 (44.0%) | |  |  |  |
|  |  |  | Macrolides | 143/663 (21.6%) | 297/1704 (17.4%) | |  |  |  |
|  |  |  | Quinolones | 221/663 (33.3%) | 662/1704 (38.8%) | |  |  |  |
|  |  |  | Tetracylines | 14/663 (2.1%) | 25/1704 (1.5%) | |  |  |  |
|  |  |  | Glycopeptides | 0 | 1/1704 (0.1%) | |  |  |  |
|  |  |  | Lincosamides | 2/663  (0.3%) | 5/1704  (0.3%) | |  |  |  |
|  |  |  | Ketolides | 30/663 (4.5%) | 53/1704 (3.1%) | |  |  |  |
|  |  |  | Co-trimoxazole | 1/663 (0.2%) | 2/1704 (0.1%) | |  |  |  |
|  |  |  | Aminoglycosides | 0 | 0 | |  |  |  |
|  |  |  | **Outpatients [**p<0.01]** | **Age <30 yrs (n=225)** | **Age ≥30 yrs (n=2180)** | | Not reported | Not reported | Not reported |
|  |  |  | Monotherapy | 232/249 (93.2%) | 1987/2118 (93.8%) | |  |  |  |
|  |  |  | Combination therapy | 15/249 (6.0%) | 124/2118 (5.9%) | |  |  |  |
|  |  |  | β-Lactams | 112/249 (45.0%) | 923/2118 (43.6%) | |  |  |  |
|  |  |  | Macrolides | 55/249 (22.1%) | 385/2118 (18.2%) | |  |  |  |
|  |  |  | Quinolones | 73/249 (29.3%)** | 810/2118 (38.2%) | |  |  |  |
|  |  |  | Tetracylines | 6/249 (2.4%) | 33/2118 (1.6%) | |  |  |  |
|  |  |  | Glycopeptides | 0 | 1/2118 (0.9%) | |  |  |  |
|  |  |  | Lincosamides | 1/249 (0.4%) | 6/2118 (0.3%) | |  |  |  |
|  |  |  | Ketolides | 14/249 (5.6%) | 69/2118 (3.3%) | |  |  |  |
|  |  |  | Co-trimoxazole | 1/249 (0.4%) | 2/2118 (0.1%) | |  |  |  |
|  |  |  | Aminoglycosides | 0 | 0 | |  |  |  |
|  |  |  | **Hospitalized patients**  **[***p<0.001, **p<0.01]** | **Age <65 yrs (n=2274)** | **Age ≥65 yrs (n=3094)** | | Not reported | Not reported | Not reported |
|  |  |  | Monotherapy | 1212/2239 (54.1%)*** | 1875/3070 (61.1%) | |  |  |  |
|  |  |  | Combination therapy | 1014/2239 (45.3%)*** | 1188/3070 (38.7%) | |  |  |  |
|  |  |  | β-Lactams | 1762/2239 (78.7%)*** | 2692/3070 (87.7%) | |  |  |  |
|  |  |  | Macrolides | 948/2239 (42.3%)*** | 1069/3070 (34.8%) | |  |  |  |
|  |  |  | Quinolones | 463/2239 (20.7%)*** | 413/3070 (13.5%) | |  |  |  |
|  |  |  | Tetracylines | 12/2239 (0.5%) | 9/3070 (0.3%) | |  |  |  |
|  |  |  | Glycopeptides | 3/2239 (0.1%) | 6/3070 (0.2%) | |  |  |  |
|  |  |  | Lincosamides | 15/2239 (0.7%) | 19/3070 (0.6%) | |  |  |  |
|  |  |  | Ketolides | 8/2239 (0.4%)** | 1/3070 (0.0%) | |  |  |  |
|  |  |  | Co-trimoxazole | 5/2239 (0.2%) | 8/3070 (0.3%) | |  |  |  |
|  |  |  | Aminoglycosides | 19/2239 (0.8%) | 12/3070 (0.4%) | |  |  |  |
|  |  |  | **Hospitalized patients [***p<0.001]** | **Age <50 yrs (n=1144)** | **Age ≥50 yrs (n=4224)** | | Not reported | Not reported | Not reported |
|  |  |  | Monotherapy | 582/1124 (51.8%)*** | 2505/4185 (59.9%) | |  |  |  |
|  |  |  | Combination therapy | 534/1124 (47.5%)*** | 1668/4185 (39.9%) | |  |  |  |
|  |  |  | β-Lactams | 854/1124 (76.0%)*** | 3600/4185 (86.0%) | |  |  |  |
|  |  |  | Macrolides | 501/1124 (44.6%)*** | 1516/4185 (36.2%) | |  |  |  |
|  |  |  | Quinolones | 248/1124 (22.1%)*** | 628/4185 (15.0%) | |  |  |  |
|  |  |  | Tetracylines | 12/1124 (1.1%)*** | 9/4185 (0.2%) | |  |  |  |
|  |  |  | Glycopeptides | 3/1124 (0.3%) | 6/4185 (0.1%) | |  |  |  |
|  |  |  | Lincosamides | 10/1124 (0.9%) | 24/4185 (0.6%) | |  |  |  |
|  |  |  | Ketolides | 5/1124 (0.4%) | 9/4185 (0.2%) | |  |  |  |
|  |  |  | Co-trimoxazole | 3/1124 (0.3%) | 10/4185 (0.2%) | |  |  |  |
|  |  |  | Aminoglycosides | 7/1124 (0.6%) | 24/4185 (0.6%) | |  |  |  |
|  |  |  | **Hospitalized patients [***p<0.001, **p<0.01]** | **Age <40 yrs (n=656)** | **Age ≥40 yrs (n=4712)** | | Not reported | Not reported | Not reported |
|  |  |  | Monotherapy | 323/663 (50.0%)*** | 2764/4661 (59.3%) | |  |  |  |
|  |  |  | Combination therapy | 317/663 (49.1%)*** | 1885/4661 (40.4%) | |  |  |  |
|  |  |  | β-Lactams | 488/663 (75.5%)*** | 3966/4661 (85.1%) | |  |  |  |
|  |  |  | Macrolides | 308/663 (47.7%)*** | 1709/4661  (36.7%) | |  |  |  |
|  |  |  | Quinolones | 137/663 (21.2%)** | 729/4661 (15.8%) | |  |  |  |
|  |  |  | Tetracylines | 8/663 (1.2%)*** | 13/4661 (0.3%) | |  |  |  |
|  |  |  | Glycopeptides | 1/663 (0.2%) | 8/4661 (0.2%) | |  |  |  |
|  |  |  | Lincosamides | 3/663 (0.5%) | 31/4661 (0.7%) | |  |  |  |
|  |  |  | Ketolides | 3/663 (0.5%) | 6/4661 (0.1%) | |  |  |  |
|  |  |  | Co-trimoxazole | 1/663 (0.2%) | 12/4661 (0.3%) | |  |  |  |
|  |  |  | Aminoglycosides | 5/663 (0.8%) | 26/4661 (0.6%) | |  |  |  |
|  |  |  | **Hospitalized patients [***p<0.001, **p<0.01]** | **Age <30 yrs (n=248)** | **Age ≥30 yrs (n=5120)** | | Not reported | Not reported | Not reported |
|  |  |  | Monotherapy | 119/241 (49.4%)** | 2968/5068 (58.6%) | |  |  |  |
|  |  |  | Combination therapy | 121/241 (50.2%)** | 2081/5068 (41.1%) | |  |  |  |
|  |  |  | β-Lactams | 190/241 (78.8%) | 4264/5068 (84.1%) | |  |  |  |
|  |  |  | Macrolides | 114/241 (47.3%)** | 1903/5068 (37.5%) | |  |  |  |
|  |  |  | Quinolones | 48/241 (19.9%) | 828/5068 (16.3%) | |  |  |  |
|  |  |  | Tetracylines | 1/241 (0.4%) | 20/5068 (0.4%) | |  |  |  |
|  |  |  | Glycopeptides | 0 | 9/5068 (0.2%) | |  |  |  |
|  |  |  | Lincosamides | 2/241 (0.8%) | 32/5068 (0.6%) | |  |  |  |
|  |  |  | Ketolides | 3/241 (1.2%)*** | 6/5068 (0.1%) | |  |  |  |
|  |  |  | Co-trimoxazole | 0 | 13/5068 (0.3%) | |  |  |  |
|  |  |  | Aminoglycosides | 2/241  (0.8%) | 29/5068  (0.6%) | |  |  |  |
| Kofteridis 2009 [30] | Adults hospitalized with community-acquired LRTI due to *H. influenza* (n=45) | Median 68 (range 28–86) | Empirical antibiotic treatment administered to all patients on admission | | | Initial empirical antibiotic treatment inappropriate in 5/45 (11%) patients. | | *H. influenzae* isolates produced β-lactamase and were resistant to amoxicillin (36/45, 80%)  Resistance to amoxicillin-clavulanate (3/45, 7%)  Resistance to ciprofloxacin (3/45, 7%) | Five patients died – all had pneumonia complicated by respiratory failure caused by strains resistant to amoxicillin |
|  |  |  |  |  |  |  |  |  |  |
|  |  |  |  |  |  |  |  |  |  |
| Kothe 2008 [23] | Patients with CAP (n=2647)  <65 yrs, n=1298 (3.5% nursing home residents)  ≥65 yrs, n=1349 (15.2% nursing home residents) | <65 yrs, mean 47.2±12.7  ≥65 yrs, mean 77.1±7.5 | Not reported | | | Patients changing antibiotic therapy due to ineffectiveness:   - <65 yrs: 141/1298, 10.9% - ≥65 yrs: 156/1349, 11.6% | | Not reported | Change of antibiotic due to treatment failure was a risk factor for 30-day mortality:   - Univariate analysis: OR 2.24 (95% CI 1.50–3.34), p=0.001 - Multivariate analysis: OR 1.74 (1.07–2.83), p=0.027   Sequential therapy (switch from i.v. to oral therapy after documentation of clinical response) related to favourable outcome for 30-day mortality:   - Univariate analysis: 0.46 (0.24–0.94), p=0.026 - Multivariate analysis: 0.26 (0.10–0.62), p=0.003 |
| Madeddu 2008 [36] | HIV patients hospitalized for CAP (n=76; 84 episodes) | Mean 38.3±7.5  (range 27–80) | Most patients received empiric antibiotic therapy, particularly with i.v. amoxicillin/clavulanic acid (76.2% of episodes) or levofloxacin (7.1%) | | | Not reported | | Not reported | Not reported |
| Manno 2009 [37] | HIV patients hospitalized for CAP  Patients with cirrhosis, n=29  Patients without cirrhosis, n=73 | Cirrhosis, mean 41.0±4.3  No cirrhosis, mean 37.3±6.2 | Cirrhotic patients:   - Levofloxacin (11/29, 38%) - Ceftriaxone (7/29, 24%) - Combination of ≥2 antibiotics (10/29, 34.5%)   Non-cirrhotic patients:   - Levofloxacin (34/73, 46%) - Clarithromycin (26/73, 35%) - Combination of ≥2 antibiotics (29/73, 39.7%)   Combination therapy more frequently based on macrolides with penicillins or cephalosporins  Fluoroquinolones (especially levofloxacin), penicillins and cephalosporins were the antibiotics more commonly used in monotherapy | | | Not reported | | Not reported | Not reported |
| Migliorati 2006 [66] | Hospitalized patients with discharge diagnosis of pneumonia or pneumonia-related disease (n=148)  (20% nursing home residents) | ≥15;  mean 70.3±17.3 | All patients received an antimicrobial treatment, more often (93%) a regimen of i.v. antibiotic therapy within 24 h of hospital admission in accordance to published guidelines (ATS 2001) | | | Not reported | | Not reported | Not reported |
| Molinos 2009 [38] | Patients hospitalized with CAP (n=710)  With COPD, n=244  No COPD, n=466  (5% nursing home residents in both groups) | Mean:  Overall, 67.14 (95% CI 65.9–68.4)  With COPD 73.7 (72.5–74.9)  No COPD, 63.6 (95% CI, 61.8–65.4) | Antibiotic therapy prior to admission  COPD patients (41/244, 17%):   - Macrolides (8/41) - Amoxicillin-clavulanic acid (14/4) - Moxifloxacin (9/41) - Levofloxacin (6/41) - Ciprofloxacin (2/41) - Cefuroxime-axetil (2/41)   No COPD (92/466, 20%):   - Amoxicillin-clavulanic acid (36/92) - Macrolides (25/92) - Moxifloxacin (10/92) - Ciprofloxacin (5/92) - Cefuroxime-axetil (5/92) - Levofloxacin (5/92) - Amoxicillin-clavulanic acid with macrolides (6/92)   Antibiotic therapy following admission:  COPD patients:   - Levofloxacin (98/244, 40%) - Amoxicillin-clavulanic acid (75/244, 31%) - Third-generation cephalosporin (16/244, 7%) - Macrolide (3/244, 1%) - Ciprofloxacin (4/244, 2%) - Third-generation cephalosporin + macrolide (25/244, 10%) - Amoxicillin-clavulanic acid + macrolide (12/244, 5%) - Third-generation cephalosporin + levofloxacin (3/244, 1%) - Amoxicillin-clavulanic acid + levofloxacin (2/244, 1%)   No COPD:   - Levofloxacin (227/466, 49%) - Amoxicillin-clavulanic acid (125/466, 27%) - Third-generation cephalosporin (24/466, 5%) - Macrolide (7/466, 1.5%) - Third-generation cephalosporin + macrolide (40/466, 9%) - Amoxicillin-clavulanic acid + macrolide (13/466, 3%) - Third-generation cephalosporin + levofloxacin (7/466, 1.5%) - Amoxicillin-clavulanic acid + levofloxacin (3/466, 1%) - Third-generation cephalosporin + clindamycin (2/466, 0.5%) - Piperacillin-tazobactam (2/466, 0.5%) | | | Not reported | | Not reported | Not reported |
| Ruiz 2010 [31] | Adults hospitalized with bacteraemic CAP due to Gram-negative bacteria (n=51) | Mean 72.9±11.3 | Not reported | | | Initial prescribed empirical antibiotic treatment inadequate in 7/51 (13.7%) patients | | Not reported | Prescription of an inadequate empirical antibiotic treatment: OR 11.0 (95% CI 1.3–96.8), p=0.031, independently associated with a severe clinical condition during hospital stay |
| Sopena 2007 [28] | Patients hospitalized with community-acquired Legionnaires’ disease (n=251)  Sporadic cases, n=138  Outbreak cases, n=113 | Mean:  Sporadic cases, 56.6±15.5  Outbreak cases, 59.5±16.6 | Sporadic   - Macrolides (77/124, 62.1%) - Quinolones (47/124, 37.9%)   Outbreak   - Macrolides (109/113, 96.4%) - Quinolones (4/113, 3.5%) | | | Adequate antibiotic treatment:   - Sporadic (124/127, 97.6%) - Outbreak (113/113, 100%) | | Not reported | Not reported |
| Sopena 2007 [29] | Patients hospitalized with CAP due to *L. pneumophila* (n=158)  <65 yrs, n=104;  ≥65 yrs, n=54 | <65, 65.9% ≥65, 34.1% ≥70, 13.9% ≥85, 1.9% | - Quinolone (43/98, 43.9%) - Macrolide (19/50, 38%) | | | Adequate antibiotic treatment   - Younger patients (98/102, 96.1%) - Older patients (50/51, 98%) | | Not reported | Not reported |
|  |  |  |  |  |  |  |  |  |  |
| Viasus 2011 [50] | Patients with and without chronic kidney disease, hospitalized with CAP (n=3800)  Kidney disease, n=203 (8.6% nursing home residents)  No kidney disease, n=3597 (8.1% nursing home residents) | Kidney disease: median 77 (IQR 67–84)  No kidney disease: median 70 (IQR 56–79) | Not reported | | | Inappropriate antibiotic therapy   - Kidney disease (3/103, 2.9%) - No kidney disease (156/2178, 7.2%) | | Not reported | Not reported |
| Viasus 2011 [46] | Patients with and without cirrhosis, hospitalized with CAP (n=3420)  Cirrhosis, n=90  No cirrhosis, n=3330 | Mean: cirrhosis, 61.8±13.0  No cirrhosis, 66.8±16.9 | Initial antibiotic therapy:  Cirrhosis   - Monotherapy (59/87, 67.8%) - Combination therapy (28/87, 32.2%)   No cirrhosis   - Monotherapy (2217/3252, 68.2%) - Combination therapy (1035/3252, 31.8%) | | | Inappropriate antibiotic therapy   - Cirrhosis (2/66, 3%) - No cirrhosis (135/1875, 7.2%) | | Pneumococcal resistance to penicillin (% of isolates):   - Cirrhosis (11.1%) - No cirrhosis (8.4%)   Pneumococcal resistance to erythromycin (% of isolates):   - Cirrhosis (15.4%) - No cirrhosis (14.7%)   Pneumococcal resistance to ciprofloxacin (% of isolates):   - Cirrhosis (0%) - No cirrhosis (1.4%)   No quinolone resistance found in *S. pneumoniae* | Not reported |
|  |  |  |  |  |  |  |  |  |  |
| Viegi 2006 [47] | Primary care patients with CAP (n=699)    Diagnosed by GP, n=548 (4.7% nursing home residents)  Diagnosed by hospital, n=151 (8.9% nursing home residents) | Patients diagnosed in community, 57.6±19.2  Patients diagnosed in hospital, 66.7±18.7  [10 patients in overall population were aged ≤14 yrs] | - Cephalosporins (45.8%) - Macrolides (20.2%) - Other β-lactams (18.6%) - Fluoroquinolones (12.2%)   Monotherapy (373/532 cases, 70.1%):   - Cephalosporins (144/532, 27.1%) - Macrolides (96/532, 18%) - β-Lactams (69/532, 13%) - Fluoroquinolones (64/532, 12%)   Combination therapy (159/532, 29.9%)   - Cephalosporins + macrolides (62/532, 11.7%) - Cephalosporins + others (18/532, 3.4%) - Cephalosporins + fluoroquinolones (17/532, 3.2%) - β-Lactams + others (12/532, 2.3%) - β-Lactams + macrolides (11/532, 2.1%) - Macrolides + others (7/532, 1.3%) - Macrolides + fluoroquinolones (5/532, 0.9%) - β-Lactams + cephalosporins (2/532, 0.4%) - β-Lactams + fluoroquinolones (2/532, 0.4%) - Cephalosporins + fluoroquinolones + others (2/532, 0.4%) - Fluoroquinolones + others (1/532, 0.2%) - β-Lactams + macrolides + fluoroquinolones (1/532, 0.2%) - β-Lactams + cephalosporins + macrolides (1/532, 0.2%) - Others (18/532, 3.4%) | | | Not reported | | Not reported | Not reported |
| Von Baum 2010 [32] | Patients with CAP (n=5130) (6% nursing home residents)  Patients with *Enterobacteriaceae* [EB], n=67  Patients with *P. aeruginosa* [PA], n=22  Patients with no definite EB/PA, n=1833 | Mean: 60±18 | Not reported | | | First-line antimicrobial therapy in patients with EB in respiratory samples and/or blood cultures:   - Adequate (25/67, 25%) - Inadequate (15/67, 22%) - Indeterminate (35/67, 53%)   In patients with definite PA pneumonia:   - Adequate (4/22, 18%) - Inadequate (14/22, 64%) - Indeterminate (4/22, 18%) | | Not reported | Not reported |

*H. influenzae, Haemophilus influenzae;* MRSA, methicillin-resistant *Staphylococcus aureus; P. aeruginosa, Pseudomonas aeruginosa; S. pneumoniae, Streptococcus pneumoniae.*

ATS, American Thoracic Society; CAP, community-acquired pneumonia; CI, confidence interval; COPD, chronic obstructive pulmonary disease; GP, general practitioner; h, hours; HIV, human immunodeficiency virus; ICU, intensive care unit; IDSA, Infectious Diseases Society of America; IQR, interquartile range; i.v., intravenous; LRTI, lower respiratory tract infection; MIC, minimum inhibitory concentration; OR, odds ratio; yr/yrs, year/years
